# A Replication-Competent Flavivirus Genome with a Stable GFP Insertion at the NS1-NS2A Junction

**DOI:** 10.3390/biology15030220

**Published:** 2026-01-24

**Authors:** Pavel Tarlykov, Bakytkali Ingirbay, Dana Auganova, Tolganay Kulatay, Viktoriya Keyer, Sabina Atavliyeva, Maral Zhumabekova, Arman Abeev, Alexandr V. Shustov

**Affiliations:** National Center for Biotechnology, Korgalzhin Hwy. 13/5, Astana 010000, Kazakhstan; tarlykov@biocenter.kz (P.T.); ingirbay@biocenter.kz (B.I.); auganova@biocenter.kz (D.A.); kulatay@biocenter.kz (T.K.); keer@biocenter.kz (V.K.); atavliyeva@biocenter.kz (S.A.); zhumabekova@biocenter.kz (M.Z.); abeev@biocenter.kz (A.A.)

**Keywords:** flavivirus, yellow fever virus, replication complex, NS1 protein, NS1-GFP fusion protein, adaptive mutations, NS1-NS2A processing

## Abstract

The flavivirus NS1 protein is a component of the viral replication complex and plays diverse, yet poorly understood, roles in the viral life cycle. To enable real-time visualization of the developing replication organelle and biochemical analysis of tagged NS1 and its interacting partners, we engineered a replication-competent yellow fever virus (YFV) replicon encoding a C-terminal fusion of NS1 with green fluorescent protein (NS1-GFP). The initial variant was non-viable in the absence of trans-complementation with wild-type NS1; however, viability was partially restored through the introduction of co-adaptive mutations in GFP (Q204R/A206V) and NS4A (M108L). Subsequent cell culture adaptation generated a 17-nucleotide frameshift within the NS1-GFP linker, resulting in a more flexible and less hydrophobic linker sequence. The optimized genome, in the form of a replicon, replicates in packaging cells that produce YFV structural proteins, as well as in naive BHK-21 cells. In the packaging cells, the adapted NS1-GFP replicon produces titers of infectious particles of approximately 10^6^ FFU/mL and is genetically stable over five passages. The expressed NS1-GFP fusion protein localizes to the endoplasmic reticulum and co-fractionates with detergent-resistant heavy membranes, a hallmark of flavivirus replication organelles. This NS1-GFP replicon provides a novel platform for studying NS1 functions and can be further adapted for proximity-labeling strategies aimed at identifying the still-unknown protease responsible for NS1-NS2A cleavage.

## Simple Summary

Viruses rely on molecular machines to replicate in living cells. The yellow fever virus and some others from fifty related viruses cause human disease, making it important to understand their replication machinery for therapeutic purposes. This machinery includes two proteins: nonstructural proteins one and two, which act as gears in a molecular clockwork. While their functions are partly understood, it remains unclear how these proteins are produced. They are generated by cleavage of a single precursor, yet the cellular “tool” responsible for this cut (a protease)—a potential antiviral target—has not been identified. In this study, we aimed to modify the precursor at an unusual site—very close to the cleavage site—by fusing nonstructural protein one to a green fluorescent protein. This forced the viral machinery to adjust by accumulating adaptive mutations. We describe the mutations in the replication machinery that allowed the modified virus to regain viability. This viral system is the first in its genus to have a large, functional foreign protein inserted within the replication machinery near the cleavage site, providing a platform to identify the elusive protease.

## 1. Introduction

Flaviviruses, members of the genus *Orthoflavivirus* within the family *Flaviviridae*, currently include 53 recognized species [1], and numerous partial flavivirus-like sequences discovered in distant animal lineages point to a vast, yet undiscovered, diversity within this group [2,3,4]. Flaviviruses are enveloped, positive-sense RNA viruses, containing a single-stranded genomic RNA approximately 11 kilobases in length. Medically important flaviviruses, such as yellow fever, dengue, Japanese encephalitis, West Nile and tick-borne encephalitis viruses, are mosquito- or tick-borne pathogens that cause significant global disease [5].

Despite the availability of vaccines for some flaviviruses, they remain a major public health threat. This necessitates a deeper understanding of still-unknown aspects of their life cycle, particularly the molecular details and structural organization of viral replication, to enable effective antiviral development.

The current understanding of flavivirus molecular biology is presented in reviews [6,7,8]. The flavivirus genome contains a single open reading frame encoding a large polyprotein. This polyprotein, with the gene order C-prM-E-NS1-NS2A-NS2B-NS3-NS4A-NS4B-NS5, is cleaved by both viral and host cellular proteases. The processing yields the structural proteins (components of the virion): capsid protein (C), pre-membrane (prM) and envelope (E), along with the non-structural proteins (NS1-5) that constitute the core viral replication machinery [9,10].

NS1 is a multifunctional, ER-lumenal glycoprotein indispensable for viral RNA replication and membrane remodeling, as well as participating in virion assembly and immune evasion [11,12,13]. NS3 acts as a serine protease (with NS2B as a cofactor), helicase and triphosphatase and also participates in viral assembly [14,15,16]. NS5 functions as an RNA-dependent RNA polymerase (RdRp) and has methyltransferase activity for RNA cap formation [17]. The transmembrane proteins NS2A, NS4A and NS4B, among other functions, anchor the complex to the ER, with NS4A inducing membrane rearrangements and NS4B forming dimers critical for replication [9,10,18,19,20].

A hallmark of flavivirus infection is the extensive remodeling of host intracellular membranes to generate characteristic vesicular and tubular structures known as replication organelles (ROs) [9,21]. These organelles house the viral replication complexes (RCs), which are established sites for viral RNA synthesis. It is widely accepted that these remodeled membranes originate from the endoplasmic reticulum (ER) [8,22,23].

The processing of the flavivirus polyprotein, which has a complex membrane topology, is known for the majority of cleavage sites [9,24,25]. Cleavages of sites located in the cytoplasm are mediated by the viral protease NS3/NS2B [14]. Cleavages of most sites within the ER lumen are performed by the host signal peptidase [26], and one additional cleavage in the sequence of prM is performed by the host proprotein convertase furin [27]. However, there still is a gap in the understanding of the mechanism of the cleavage at one site, the NS1-NS2A junction [28]. This cleavage is essential for the viral life cycle, as it liberates the functional proteins NS1 and NS2A from the precursor polyprotein NS1-NS2A. This cleavage is currently understood to be mediated by an unknown host cell protease resident within the endoplasmic reticulum (ER). The substrate requirement for this cleavage event is unusual; it necessitates an octapeptide motif (L/M-V-X-S-X-V-X-A) at the NS1 C-terminus as well as the presence of a substantial portion of the NS2A sequence [11,29,30,31].

The cleavage of the NS1-NS2A precursor is critical, as mature NS1 and NS2 play essential roles in viral replication. The NS1 protein is co-translationally translocated into the endoplasmic reticulum (ER) lumen via the signal peptide at the C-terminus of the preceding envelope (E) protein [11]. The mature, glycosylated form of NS1 has an apparent molecular weight of 50–55 kDa. The mature NS1 protein has a crystallographically confirmed three-domain architecture—an N-terminal β-roll, a central α/β wing and a C-terminal β-ladder—and exists as a stable dimer [13]. Despite lacking transmembrane regions, a fraction of NS1 remains associated with the ER membrane, decorating its luminal leaflet, where it is thought to induce membrane remodeling. Another fraction is secreted into the extracellular space as hexamers or tetramers. The membrane-remodeling activity induces positive curvature (invagination into the lumen), forming the vesicles that house the viral replication complexes. Despite NS1 having properties atypical for a component of the replication complex, this protein is indeed indispensable for the initiation of minus-strand RNA synthesis [32,33].

NS2A is a ~24 kDa integral membrane protein with five predicted transmembrane helices. Crystallographic structures of flavivirus NS2A are currently unavailable; consequently, all understanding of its transmembrane topology is derived from predictive modeling and biochemical experiments [34]. One of NS2A’s known functions is to coordinate virion assembly [35,36,37]. NS2A recruits nascent viral RNA; this is mediated by an interaction between a cytosolic loop of NS2A and the 3′UTR of the genome [36,38]. NS2A is therefore believed to transport the nascent genomic RNA from the replication complex to the virion budding site. NS2A also recruits the structural polyprotein C-prM-E and the NS2B-NS3 protease to this site, presumably to coordinate C-prM cleavage and nucleocapsid formation [37,39]. However, a detailed mechanistic understanding of these functions remains lacking.

Attempts to identify the protease responsible for cleaving the NS1/NS2A junction through analysis of biochemical properties, comparison to known proteases and mutational analysis of the cleavage site have been unsuccessful [29]. Future approaches may therefore rely on in vivo proximity labeling of this elusive enzyme, followed by affinity capture and mass spectrometric identification of the labeled proteome.

Proximity labeling is a method for analyzing protein interactions. It involves genetically fusing a promiscuous biotinylating enzyme (e.g., TurboID, miniTurbo) to a “bait” protein. This fusion enzyme attaches small tags to nearby proteins in living cells, which are then captured and identified by mass spectrometry [40,41]. For example, to identify the protease in question, the labeling enzyme can be fused to the C-terminus of NS1. To preserve the essential native cleavage sequence, the replicating RNA would be designed as NS1-X-NS1′-NS2A, where “X” is the labeling enzyme and “NS1′” is a short C-terminal fragment of NS1 containing the critical residues for proteolysis. This design positions the labeling enzyme in a spatial proximity to the cleavage site, enabling it to transfer a biochemical tag (e.g., biotin) to the protease if the enzyme’s activation window coincides with the proteolytic event.

However, the capacity of flavivirus NS-proteins to incorporate large heterologous sequences appears limited, likely due to the conformational sensitivity and essential roles. The development of functional, genetically tagged versions of NS1 or NS2A within a replicating genome has proven challenging, as evidenced by the scarcity of successful reports [39,42,43].

Building on our previous success in creating a replication-competent YFV with N-terminally tagged NS1 (GFP-NS1) [42], this study aims to engineer a C-terminally tagged NS1-GFP fusion that remains functional within replicating RNA. The resulting replicon or recombinant replicable genome would serve to probe the structural constraints of this critical domain and to create a platform for future substitution of GFP with a proximity-labeling enzyme such as TurboID.

## 2. Materials and Methods

### 2.1. Cell Culture

Baby Hamster Kidney (subclone C-13, ATCC CCL-10, Manassas, VA, USA) cells were cultured in Dulbecco’s Modified Eagle Medium (DMEM, high glucose, Gibco Cat. 11965092, Grand Island, NY, USA) supplemented with 10% fetal bovine serum (FBS, Gibco Cat. 10091148, Auckland, New Zealand), 1% penicillin/streptomycin (Gibco, Waltham, MA, USA) and 2 mM L-glutamine (Gibco, Waltham, MA, USA) at 37 °C in a 5% CO_2_ humidified atmosphere.

### 2.2. Construction of Molecular Infectious Clones (MICs)

All viral replicons and minigenomes were engineered as DNA-launched molecular infectious clones. Replicons and minigenomes were cloned downstream of the human cytomegalovirus (CMV) immediate-early promoter. The antigenomic ribozyme of the hepatitis delta virus (HDV-Rz) and the polyadenylation signal (PA) from the human growth hormone (HGH) gene were placed downstream of the viral 3′ terminus to ensure proper RNA processing and nuclear export.

Standard molecular cloning techniques were used. Plasmid DNA was isolated by the alkaline lysis method and further purified for transfections by banding in cesium chloride gradients.

### 2.3. Yellow Fever Virus (YFV) Replicons and Minigenomes

Yellow fever virus (YFV) sequences were derived from the 17D vaccine strain. The enhanced green fluorescent protein (eGFP) gene was amplified from plasmid pcDNA3-EGFP (Addgene Cat. 13031). A fused sequence encoding GFP followed by the 2A peptide from Foot-and-Mouth Disease Virus (FMDV) was developed (gene cassette GFP-2A).

**Figure 1:**
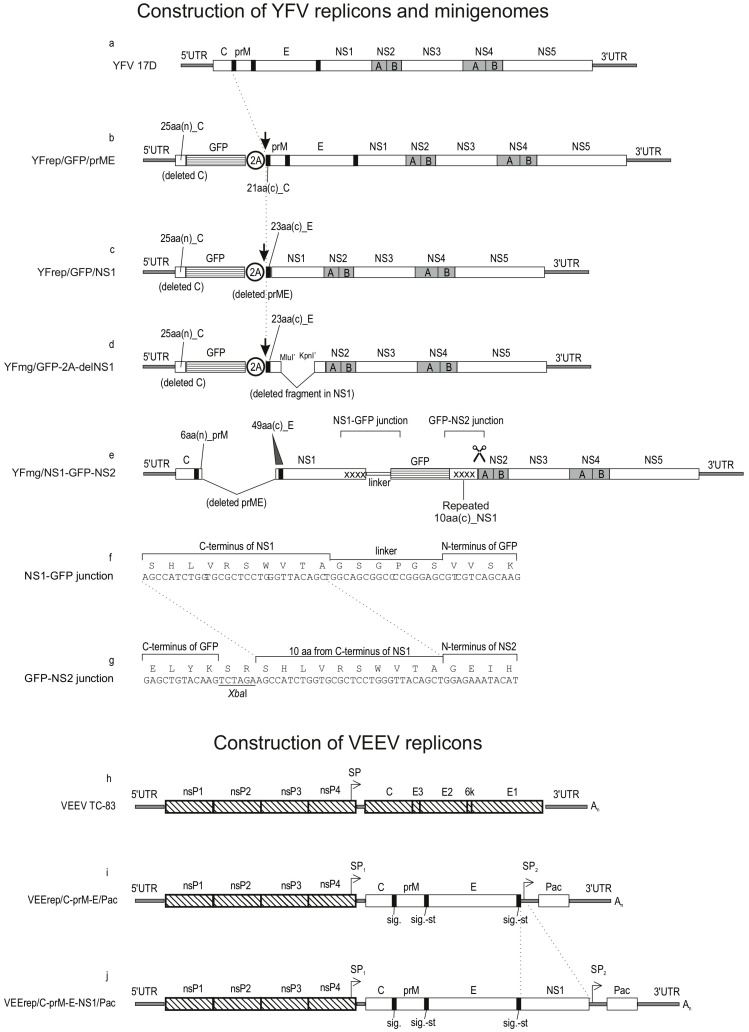
Schematic representation of VEEV-based packaging and complementing helpers and YFV replicons and minigenomes. (**a**) Genome organization of the yellow fever virus (YFV), shown for comparison with the derivatives created in this study. Genes for functional proteins are shown as rectangles, with the NS2 and NS4 genes shaded. Hydrophobic sequences present at the C-termini of the C, prM and E proteins are depicted as black rectangles. UTR, untranslated region. (**b**) YFV replicon YFrep/GFP/prME, featuring a deletion of the major part of the C gene and an insertion of GFP followed by a 2A peptide and the prM–E genes. Retained short fragments of the C gene are indicated: 25aa(n)_C, encoding 25 amino acids (a.a.) from the N-terminus; 21aa(c)_C, encoding 21 a.a. from the C-terminus. The vertical arrow designates the site of autoproteolytic cleavage mediated by the 2A peptide (its position is indicated by the open circle in the schematic). (**c**) YFV replicon YFrep/GFP/NS1, which lacks the entire structural proteins C-prM-E. The GFP-2A segment is fused to the last 23 a.a. of the E protein, which serves as a signal peptide for NS1 translocation. (**d**) YFV minigenome YFmg/GFP-2A-delNS1, which lacks the C-prM-E genes and contains a deletion in the NS1 gene. The deletion is located between the blunted MluI and KpnI restriction sites (denoted as MluI′ and KpnI′ in the schematic). (**e**) YFV minigenome YFmg/NS1-GFP-NS2, which expresses an NS1-GFP fusion protein. The NS1 and GFP domains are connected via a short peptide linker. A 10-a.a. sequence from the C-terminus of NS1 is duplicated at the C-terminus of GFP and precedes the NS2 gene to preserve authentic proteolytic processing at the newly formed NS1-NS2 junction. A fragment within the prM-E genes was deleted, leaving only six a.a. from the N-terminus of prM fused to 49 a.a. from the C-terminus of E. (**f**,**g**) Nucleotide and amino acid sequences of the junctions for the NS1-GFP (**f**) and GFP–NS2A (**g**) fusions. A 6-a.a. linker is present at the NS1-GFP junction. The GFP gene is fused to the sequence encoding the ten C-terminal a.a. of NS1 via a linker containing an *Xba*I site (underlined). The scissors sign indicates that the authentic proteolytic processing site for the NS1/NS2 cleavage is preserved at the GFP-NS2 junction. (**h**) Genomic organization of the Venezuelan equine encephalitis virus (VEEV), presented for comparison. Genes for functional proteins are shown as rectangles. VEEV-specific genes are indicated by hatched shading. The VEEV subgenomic promoter is depicted as a right-ward arrow. Designations: UTR, untranslated region; nsP, nonstructural proteins; SP, subgenomic promoter; C-E3-E2-6k-E1, VEEV polyprotein for structural proteins; An, oligo-A stretch terminating the VEEV genome. (**i**) VEEV replicon engineered to express a gene cassette for YFV capsid (C) and prM–E. The gene cassette is under the control of the first copy of the subgenomic promoter (SP1). The second copy of the subgenomic promoter (SP2) controls the puromycin resistance gene (Pac). YFV-specific genes and the Pac gene are shown as open rectangles. Elements: sig., signal peptide for prM translocation; sig.-st, signal peptide and stop-transfer sequence at the C-termini of the prM and E proteins. (**j**) VEEV replicon engineered to express YFV C-prME-NS1.

YFrep/GFP/prME is a replicon featuring a deletion of most of the capsid (C) gene. A 75-nucleotide fragment from the 5′ end of the capsid (C) gene is retained because it contains the conserved 5′ cyclization sequence (5′-CS), a cis-acting element essential for replication. The encoded 25 a.a.-long peptide from C is fused to the GFP-2A protein. The same genetic organization (25C-GFP-2A) was copied across all YFV variants described in this study, except one minigenome (Figure 1). In YFrep/GFP/prME, the C-terminus of the 2A peptide is followed by the 21 C-terminal amino acids (a.a.) of the C protein, which serve as the signal peptide for prM-E translocation.

YFrep/GFP/NS1 is a replicon in which the entire structural protein region (C-prM-E) is replaced by a GFP-2A cassette. The GFP-2A segment is fused to the C-terminal 23 a.a. of the E protein, which serves as a signal peptide for NS1 translocation.

YFmg/GFP-2A-delNS1 is a minigenome that lacks the C-prM-E genes (replaced with GFP-2A cassette) and contains a deletion in the NS1 gene. GFP-2A is fused in-frame to the C-terminal 23 a.a. of the E protein. A large in-frame deletion within the NS1 gene was generated by digesting the parental NS1 sequence with MluI and KpnI restriction enzymes, blunting the resulting DNA fragment ends with a mixture of Klenow fragment and T4 DNA polymerase and re-ligating the blunted ends, thereby removing the intervening NS1 sequence.

YFmg/NS1-GFP-NS2 is a minigenome, engineered to produce a fusion protein with GFP attached to the C-terminus of NS1. This minigenome contains the full-length capsid (C) gene but carries a deletion within the prM-E region, retaining only six N-terminal a.a. of prM and 49 C-terminal a.a. of the envelope (E) protein. The GFP gene was inserted into the intergenic region between NS1 and NS2A to create a stable NS1-GFP fusion. A 6-a.a. linker (Gly-Ser-Gly-Pro-Gly-Ser) connects NS1 to GFP. The native start codon (ATG) of the GFP gene was replaced with a valine codon (GTG) to engineer an *Aat*II restriction site for cloning convenience. This Met-to-Val modification at the GFP N-terminus did not measurably affect GFP fluorescence intensity, as confirmed in preliminary experiments. This same Val-initiated GFP sequence was used in all variants constructed before obtaining the culture-adapted variant with a spontaneous rearrangement affecting the beginning of GFP (e.g., YFVmg-v2/GFP*, YFVmg-v5/GFP* (NS4*), etc.).

To ensure authentic proteolytic cleavage at the NS1-NS2A junction, a GFP gene was fused in-frame to the C-terminal 10 a.a. of NS1 via an engineered *Xba*I restriction site. This NS1-GFP cassette was then inserted upstream of the NS2A gene.

### 2.4. Venezuelan Equine Encephalitis Virus (VEEV) Helper Replicons

Two VEEV (TC-83 strain) replicons were constructed (Figure 1). These replicons retain the VEEV nonstructural genes (nsP1–4) and contain duplicated subgenomic promoters (SPs) for transgene expression. The genetic design of the VEEV replicons is similar to that previously described [44].

The replicon VEErep/C-prM-E/Pac, or packaging helper, expresses YFV structural proteins C-prM-E and puromycin acetyltransferase (Pac). The first SP copy directs the synthesis of a subgenomic RNA encoding the YFV C-prM-E polyprotein. The second SP drives synthesis of the subgenomic RNA for the puromycin resistance marker, Pac.

The replicon VEErep/C-prM-E-NS1/Pac, or complementing helper, expresses YFV proteins C-prM-E-NS1 and Pac. Its genetic organization is similar to the packaging helper, but the YFV-specific coding region is extended to include the complete NS1 gene.

### 2.5. Generation of Stable Packaging and Complementing Cell Lines

BHK-21 cells were transfected with the VEEV helper RNAs using electroporation (see Section 2.6). At 24 h post-transfection, the culture medium was replaced with medium containing 10 µg/mL puromycin to select stable cell lines. Puromycin-resistant (Pur+) cells were expanded under continuous selection and cryopreserved. Thus, the packaging cell line (BHK/VEErep/C-prM-E/Pac) and the complementing cell line (BHK/VEErep/C-prM-E-NS1/Pac) were established.

### 2.6. Transfection for YFV Replicon/Minigenome Rescue

Cells were grown to ~90% confluence in P150 dishes, harvested and used for electroporation. Cells (1 × 10^7^) were mixed with 10 µg of plasmid DNA in a 2 mm gap cuvette and electroporated using a Gene Pulser II apparatus (Bio-Rad, Hercules, CA, USA) with two exponential-decay pulses (750 V, 25 µF, infinite resistance). Cells were recovered for 10 min at room temperature and transferred to P100 dishes in complete medium. Electroporations were performed in triplicate.

### 2.7. Passaging Infectious Particles, Focus Purification

Cell passaging: transfected cultures reaching near-confluence were split (1:5) into new P100 dishes.

Passaging of infectious particles: if a minigenome is capable of packaging into infectious particles, these can be serially passaged in complementing or packaging cells, analogous to a live virus. To initiate a new passage, fresh monolayers (~2 × 10^6^ cells in a P100 dish) were inoculated with the designated volume of stock from the previous culture: 1 mL for uncloned/unpurified particle stocks or 100 µL for focus-purified particle stocks.

In this study, it was important to distinguish between YFV-derived GFP-expressing variants capable of producing infectious particles and those producing only sporadic GFP-positive cells upon transfection of the corresponding MICs. Therefore, a variant was operationally defined as replication- and packaging-competent if its culture supernatant contained infectious particle titers above the assay’s limit of detection (50 FFU/mL) and the percentage of GFP+ cells increased over time, evidenced by microscopy or quantified by flow cytometry, over a 72 h period post-transfection/infection. Conversely, a variant was classified as incapable of spreading infection if infectious particle titers were below the limit of detection and the percentage of GFP+ cells declined or failed to increase significantly over the same period, a result attributable to the overgrowth of uninfected cells.

Focus purification: packaging cells were seeded in 6-well plates at a density of 5 × 10^5^ cells per well. Following adherence, the monolayers were infected with serial tenfold dilutions of the particle stock. Infected monolayers were then overlaid with a semi-solid medium consisting of a 1:1:1 mixture of 1× complete DMEM, 2× EMEM (without additives), and 2% low-melting-point agarose in water. After an incubation period of 3–5 days, well-separated fluorescent foci (~2–5 mm in diameter) were visualized under a fluorescence microscope. The agar overlay above the selected foci was excised, and the agar plugs were transferred to 1 mL of PBS containing 1% horse serum. Particles were eluted overnight at 4 °C. The eluted variants were then amplified by infecting fresh cell cultures.

### 2.8. Titration of Infectious Particles

Focus-forming assay was performed in packaging or complementing cells. 90%-confluent monolayers in 6-well plates were inoculated with serial dilutions of the particle stock. After adsorption, the cells were overlaid with agar-containing medium. GFP-positive foci were visualized and counted 3–5 days post-infection. Titers were expressed as focus-forming units per milliliter (FFU/mL).

The limit of detection (LOD) for the focus-forming assay was determined based on the lowest dilution of the sample assayed and the volume of inoculum applied. For a standard assay using 200 µL of inoculum per well in a 6-well plate, the LOD was 50 FFU/mL.

### 2.9. Flow Cytometry Analysis

The percentage of GFP-positive (GFP+) cells was quantified by flow cytometry. Cells were detached using trypsin-EDTA, washed with phosphate-buffered saline (PBS), resuspended in MACSQuant Running Buffer (Miltenyi Biotec Cat. 130-092-747, Bergisch Gladbach, Germany) and passed through a 70 µm strainer. Samples were analyzed on a MACS Quant Analyzer 10 flow cytometer (Miltenyi Biotec, Bergisch Gladbach, Germany). Data were processed using MACSQuantify Software 3.0.2 (Miltenyi Biotec). Debris was excluded by gating on the forward-scatter versus side-scatter (FSC-A vs. SSC-A) plot. Single cells were gated using the forward-scatter height versus forward-scatter area (FSC-H vs. FSC-A) plot. GFP fluorescence was measured in the channel B1 488/(530/30) nm. The reported percentages of GFP+ cells are based on the following gating strategy. Gates were set up using untransfected BHK-21 cells as a negative control population, so that the GFP+ gate contained 0.1% of events; this gate was then applied to all experimental samples. The GFP+ gate was validated using a cell population with near-universal GFP expression. For this purpose, BHK-21 cells were infected (MOI, 10) with a fast-propagating recombinant GFP-expressing virus described previously [42], which results in nearly all cells becoming GFP-positive within 36 h post-infection, prior to the accumulation of dead cells. Additionally, commercial calibration particles (Miltenyi Biotec, Cat. 130-093-607), composed of a mixture of fluorescent and non-fluorescent particles, were used to verify gate placement. These calibration particles were routinely analyzed before each experiment to ensure measurement stability.

### 2.10. Sequencing of Focus-Purified Replicon Genomes

Total RNA was extracted from infected cells using TRIzol. Half of the total RNA obtained from a P100 dish was converted to cDNA using Superscript II reverse transcriptase and random hexamers or YFV-specific primers. PCR was performed with high-fidelity DNA polymerase Phusion. PCR products were purified and sequenced directly by Sanger sequencing using a set of overlapping primers.

For sequencing characterization of adapted variants, amplicons covering the region homologous to 164–10,534 nt in the YFV 17D genome (GenBank Acc. X03700.1), as well as the complete heterologous inserts, were generated and sequenced. This region encompasses the nearly complete structural protein genes (C-prM-E), all the non-structural genes (NS1-NS5), the inserted GFP gene and all engineered junctions. The 5′- and 3′-terminal ~160 nucleotides, consisting primarily of conserved untranslated regions (UTRs), were not routinely sequenced because they were not modified in our constructs. To ensure clonal purity before sequencing, each adapted variant was subjected to focus purification; replicating RNAs from three independently isolated, large fluorescent foci were individually amplified and sequenced. The resulting three full-genome contigs were then aligned and compared to detect any differences between the individually sequenced clones and to confirm the homogeneity of the adapted population.

### 2.11. Analysis of Replication Kinetics and Genetic Stability

Replication kinetics: packaging cells were transfected or infected in triplicate cultures. Culture supernatant samples were collected daily and titrated.

For the purpose of analyzing the genetic stability of the YFrep/NS1-GFP replicon, the NS1-GFP-NS2A region was sequenced from the passage P5. Infectious particles from passage P5 were pelleted by ultracentrifugation (140,000× *g* for 2 h at 4 °C; Optima MAX-XP ultracentrifuge (Beckman Coulter, Indianapolis, IN, USA) with an MLA-130 rotor (Beckman Coulter, Indianapolis, IN, USA)), followed by RNA extraction, reverse transcription and PCR amplification using primers targeting NS1-GFP-NS2A. The resulting amplicons were cloned into the pGEM-T vector (Promega Cat. A3600). The plasmid clones were sequenced, ensuring that each part in the NS1-GFP-NS2A region was covered by sequences from at least ten independent clones.

### 2.12. Western Blot Analysis

Packaging cells in P100 dishes were infected with YFrep/NS1-GFP replicon particles at an MOI = 1. A parallel culture of packaging cells was infected with the YFrep/GFP/prME replicon (MOI = 1). Cells were incubated for 48 h. Cells were detached by trypsinization, collected into 15 mL conical tubes and pelleted by centrifugation (500× *g*, 5 min). Cell pellets were washed once with ice-cold PBS and lysed in 2× Laemmli protein loading buffer. Control antigens included a lysate prepared from naive BHK-21 cells and an inactivated lysate from BHK-21 cells infected with YFV 17D from the laboratory repository. All lysates were boiled (100 °C) for 3 min before electrophoresis. Proteins were resolved by SDS-PAGE on 10% polyacrylamide gels and transferred onto nitrocellulose membranes (Protran, Amersham, UK).

Membranes were blocked for 1 h at room temperature in PBS-T (PBS with 0.1% Tween 20) containing 5% non-fat dry milk and 1 mg/mL bovine serum albumin. For immunodetection, membranes were incubated with primary antibodies as described in the figure legends. YFV antigens were detected using mouse anti-YFV antiserum (laboratory repository) at a 1:200 dilution; YFV NS1 was specifically detected using a mouse monoclonal anti-YFV NS1 antibody (Abcam ab253237) at a 1:500 dilution. GFP was detected using a DyLight800-conjugated goat anti-GFP antibody (Rockland Immunochemicals, 600-145-215) at a 1:5000 dilution.

The membranes were washed in PBS-T. Bound anti-YFV mouse antibodies were detected using a donkey anti-mouse IgG secondary antibody conjugated to a 680 nm fluorophore (IRDye 680RD, LI-COR Biotechnology Cat. 926-68072) at 1/20,000 dilution. The membrane was scanned using an Odyssey DLx infrared imaging system (LI-COR Biotechnology, Lincoln, NE, USA). The scanning was performed at 169 µm resolution with laser intensities set to 6.0 for the 700 nm channel (detecting anti-YFV antibodies) and 6.0 for the 800 nm channel (detecting anti-GFP antibodies). The limit of detection was empirically determined by using a dilution series of a purified recombinant GFP standard (ThermoScientific, Cat. A42613, Waltham, MA, USA). Under these settings (169 µm resolution, laser intensity 6.0), Odyssey DLx reliably detects a band containing 1 ng of GFP.

### 2.13. Isolation of Heavy Membranes

For the isolation of heavy membranes, naive BHK-21 cells (not containing VEEV helpers) were transfected with the YFrep/NS1-GFP replicon MIC using Lipofectamine 2000 (Invitrogen Cat. 11668019, Carlsbad, CA, USA) according to the manufacturer’s instructions. Transfection efficiency was monitored via GFP fluorescence, and cells were harvested upon reaching a strong fluorescent signal. Cells from six P150 dishes were washed with ice-cold PBS, detached using trypsin-EDTA and trypsin was inactivated with complete growth medium. The cell suspension was pooled and pelleted by centrifugation (600× *g*, 5 min, 4 °C), followed by one wash with PBS.

The cell pellet was resuspended in 1 mL of lysis buffer (1% Triton X-100 in TNMg buffer: 10 mM Tris, pH 8.8, 10 mM sodium acetate, 1.5 mM MgCl_2_). Complete lysis was achieved by homogenizing the suspension with 10 strokes in a Dounce homogenizer, followed by 10 passages through a 22-gauge needle. The lysate was incubated on ice for 1 h and then clarified by sequential centrifugation: first at 720× *g* for 5 min (4 °C) to pellet nuclei (P720), and then the resulting supernatant was centrifuged at 16,000× *g* for 15 min (4 °C) to pellet the heavy membrane fraction (P16).

The P16 pellet, containing replicative complexes, was resuspended in 0.5 mL of TNMg buffer. This suspension was mixed with 4 mL of 75% (*w*/*v*) sucrose in TNMg buffer. A discontinuous sucrose gradient was prepared in a 14 × 89 mm tube (SW41Ti rotor, Beckman Coulter, Indianapolis, IN, USA) by sequentially layering from the bottom: 0.5 mL of 75% sucrose, 4.5 mL of the P16/75% sucrose mixture, 4 mL of 55% sucrose and finally TNMg buffer containing 5% sucrose to fill the tube. Ultracentrifugation was performed at 148,000× *g* (35,000 rpm in an SW41Ti rotor, Beckman Coulter, Indianapolis, IN, USA) for 18 h at 4 °C.

Following centrifugation, the gradient was manually fractionated from the top into thirteen 1 mL fractions. Proteins from all samples were subjected to separation in 10% SDS-PAGE and visualized by Coomassie Brilliant Blue staining. The visible protein bands were excised from the gel and subjected to protein identification by tryptic peptide mass spectrometry.

### 2.14. Statistical Analysis

Transfections to determine titers of infectious particles were performed in triplicate. Data were analyzed in GraphPad Prism version 9.3.1 (GraphPad Inc., San Diego, CA, USA). The titers were log-transformed and used to compute statistical significance (*p*-value) for differences between means using an unpaired two-tailed Student’s *t*-test. Symbols to denote *p*-values were the following: ns (*p* > 0.05); * (*p* ≤ 0.05); ** (*p* ≤ 0.01); *** (*p* ≤ 0.001). Graphs for titers show arithmetic means and standard deviations (SDs).

## 3. Results

### 3.1. YFV Variants, VEEV Helpers and Results of Transfection into Trans-Complementing Cell Lines

Figure 1 (panels a–g) shows the genetic organization of the YFV replicons and minigenomes created for the initiation of this work. All viral replicons and minigenomes described herein were engineered as DNA-launched molecular infectious clones (MICs). For clarity in describing the experiments and their outcomes, we use the following terms throughout the Results and Discussion sections:

Molecular infectious clone (MIC) is a plasmid in which a viral genome or a genome-derived construct is placed under the control of a strong eukaryotic promoter, such as the CMV promoter. Downstream of the genomic 3′ terminus, genetic elements are included to ensure proper processing of the 3′ end. Upon transfection of MIC DNA into cell culture, transcription, processing and nuclear export of the transcripts lead to the appearance of viral replicons or minigenomes in the cytoplasm.

Replicon: An RNA molecule capable of self-replication (encoding a functional replication machinery, NS1-NS5) but lacking one or more structural protein genes (e.g., C-prM-E). Replicons produce replicon RNA intracellularly but require structural proteins supplied in trans to form infectious particles.

Minigenome: A replication-deficient viral RNA lacking an essential part of the replication machinery (e.g., a deletion in NS1). Minigenomes require trans-complementation with the missing protein(s) for RNA amplification.

Infectious Particles (or Packaged Replicon Particles): Virions containing replicon RNA, produced when structural proteins are supplied in trans. These particles are infectious and can be serially passaged in complementing cells, but they deliver only the replicon (lacking structural genes) to newly infected cells. Minigenomes, which lack autonomous replication, can theoretically be packaged only if the missing replication protein (e.g., NS1) is supplied in trans within the same producer cell.

YFV genome: refers to the complete, wild-type YFV 17D sequence, used for comparison.

The replicon YFrep/GFP/prME (Figure 1b) lacks the capsid protein (C) gene but retains the envelope protein genes prM and E. In place of the capsid gene, a reporter cassette ‘GFP-2A peptide’ is inserted. To ensure replication competence, the cyclization signal (5′-CS) is preserved. In the genome of mosquito-borne flaviviruses, the 5′-CS sequence is located within the fragment of the C gene encoding the first 25 a.a. of the capsid protein.

The ribosome-skipping activity of the 2A peptide in the reporter protein cleaves the GFP-2A moiety from the viral polyprotein, which begins with the prM export signal (i.e., C-terminal 21 a.a. of C). With this genetic design, GFP is produced in the cytoplasm. For infectious propagation in cell culture, the YFrep/GFP/prME replicon requires an external (in trans) source of the C protein.

The replicon YFrep/GFP/NS1 (Figure 1c) has the entire structural protein region replaced with a reporter cassette ‘GFP-2A’. The viral polyprotein begins with the last 23 a.a. of the E protein, which serve as the signal peptide for translocating NS1 into the lumen of the endoplasmic reticulum (ER). This replicon produces GFP in the cytoplasm. For infectious propagation in cell culture, YFrep/GFP/NS1 requires an external source of structural proteins (C, prM, E).

The minigenome YFmg/GFP-2A-delNS1 (Figure 1d) lacks structural protein genes and contains a major deletion in the NS1 gene, thereby precluding self-sustained replication. A reporter cassette was cloned in place of the missing C-prM-E genes. This minigenome produces GFP in the cytoplasm. The 2A peptide serves to cleave GFP from the viral polyprotein, which begins with the signal peptide for translocating NS1 into the ER lumen.

A different minigenome, YFmg/NS1-GFP-NS2 (Figure 1e–g), retains all structural protein genes and contains sequences encoding all non-structural proteins. The essential modification in this minigenome is the insertion of the GFP gene into the intergenic region between the NS1 and NS2A genes, resulting in the genetic fusion NS1-GFP. The linker between NS1 and GFP was designed to lack cleavage sites for known proteases (Gly-Ser-Gly-Pro-Gly-Ser). To preserve the authentic proteolytic processing site after GFP insertion, a duplicated 10-a.a. sequence from the C-terminus of NS1 was introduced following GFP, thus maintaining the known processing requirements at the NS1-NS2 junction.

Two VEEV (strain TC-83) replicons were engineered to enable trans-complementation of the replication-deficient YFV RNAs (Figure 1h–j). The VEEV replicons retain all sequences enabling autonomous RNA replication and subgenomic mRNA transcription, namely, the 5′ and 3′ UTRs and the genes for non-structural proteins (nsP1–4). The genes encoding the VEEV structural proteins (C–E3–E2–6k–E1) have been deleted and replaced with YFV-derived genes. Additionally, a second copy of the subgenomic promoter (SP) was inserted downstream of the YFV-derived genes, creating a new cistron designed to express a selection marker. In the resulting replicons, the first SP copy drives the synthesis of the YFV protein-encoding subgenomic RNA, while the second SP copy directs the synthesis of a different subgenomic RNA, encoding puromycin resistance marker (puromycin acetyltransferase, Pac). These VEEV replicons were cloned into the respective MICs.

One replicon VEErep/C-prM-E/Pac (Figure 1i) is designed to provide the YFV structural proteins (C-prM-E) required for the assembly of infectious particles that encapsidate YFV replicons. Another replicon VEErep/C-prM-E-NS1/Pac (Figure 1j) supplies not only the YFV structural proteins but also the non-structural protein NS1, which is essential to trans-complement the replication of YFV minigenomes.

To generate cell lines harboring VEEV replicons, BHK-21 cells were transfected with the VEEV MICs. Twenty-four hours post-transfection, puromycin was added to the culture medium. Within 24 h of puromycin addition, a significant portion of cells had died; however, surviving (Pur+) cells were also present. The Pur+ culture resumed growth, and by 72 h after puromycin selection, it could be passaged into larger culture flasks. The Pur+ cultures were subsequently expanded and cryopreserved for long-term storage.

**Figure 2:**
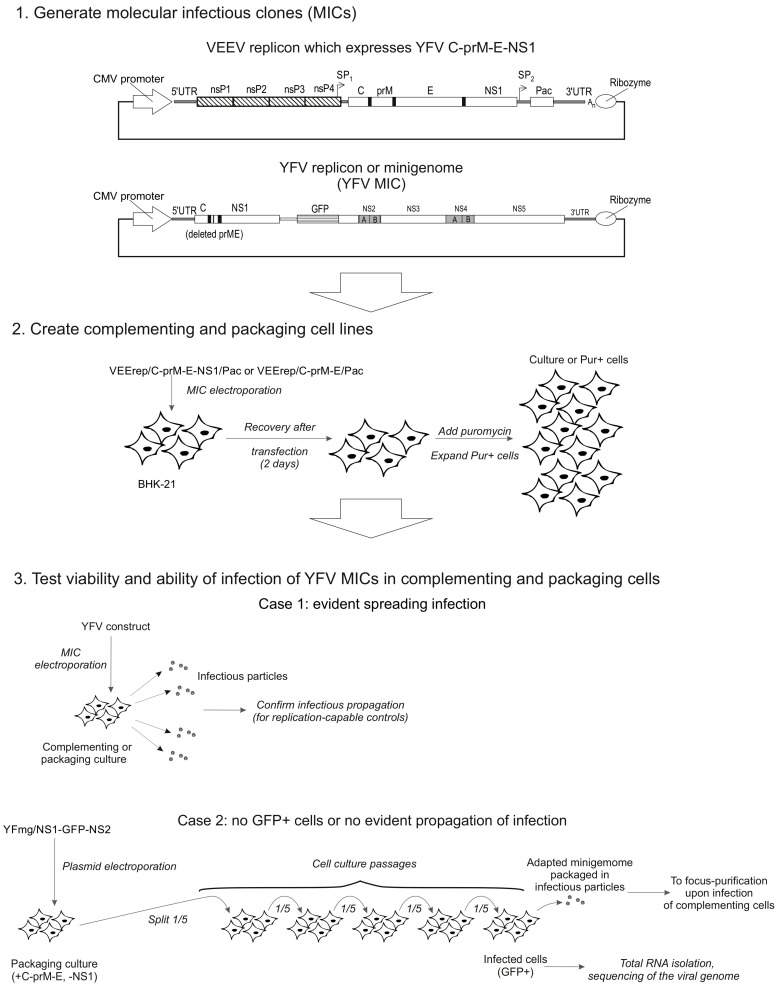
Planned experimental strategy for the rescue and adaptation of the YFV NS1-GFP-NS2 minigenome. The strategy involves the following steps: (**1**) Generation of molecular infectious clones (MICs) to achieve cytoplasmic replication of minigenomes or replicons. Only two MICs are shown as examples out of four YFV RNAs and two VEEV helpers. One of the shown VEEV replicons expresses the YFV proteins C-prM-E-NS1. The shown YFV minigenome YFmg/NS1-GFP-NS2 has GFP fused to the C-terminus of NS1. (**2**) Creation of complementing and packaging cell lines. BHK-21 cells are transfected with VEEV MICs to harbor either the VEErep/C-prM-E-NS1/Pac replicon (complementing cells) or the VEErep/C-prM-E/Pac replicon (packaging cells), and a population of puromycin-resistant cells is selected. (**3**) Testing of YFV variants for the capability of replication and infectious propagation. YFV MICs are electroporated into the established cell lines. A successful rescue (Case 1) was defined by the production of infectious particles at titers above the limit of detection (LOD, 50 FFU/mL) and an increase in the percentage of GFP+ cells over the 72 h observation period, as quantified by flow cytometry. If neither measurable titers nor an increase in GFP+ cells was observed by 72 h post-transfection (Case 2), the culture was subjected to serial passaging. This strategy provided an opportunity for slowly replicating, autonomously replicating RNAs to accumulate adaptive mutations that could restore packaging competence and propagation. Emerging adapted variants are subsequently subjected to focus purification and sequencing. Designations: CMV, cytomegalovirus immediate-early promoter; Pac, puromycin N-acetyltransferase.

The following experimental strategy was planned (Figure 2). After constructing all planned MICs, two types of trans-complementing cell lines were established upon MIC transfection and puromycin selection: a packaging cell line, consisting of BHK-21 cells harboring the VEErep/C-prM-E/Pac replicon; and a complementing cell line, consisting of BHK-21 cells harboring the VEErep/C-prM-E-NS1/Pac replicon.

Four YFV MICs were transfected into trans-complementing cells: YFrep/GFP/prME, YFrep/GFP/NS1, YFmg/GFP-2A-delNS1 and YFmg/NS1-GFP-NS2. Variants were considered successfully rescued (Case 1, Figure 2) if they showed increasing GFP+ cells and produced infectious particles. For non-viable variants (Case 2, Figure 2), lacking both criteria, the transfected cultures were serially passaged. This provided an opportunity for the slowly replicating RNA to accumulate adaptive mutations that increase its capacity for autonomous replication and restore packaging into infectious particles.

We expected all four YFV MICs to be viable in complementing cells, as this cell line supplies NS1 for replication and C-prM-E for packaging. However, it was possible that YFV minigenomes would not initiate cytoplasmic replication or would replicate poorly in packaging cells, due to the absence of NS1 supplied in trans. The possibility was reserved that accumulating spontaneous substitutions in a slowly replicating self-replicating RNA could lead to the emergence of adapted variants. If such variants capable of efficient propagation arose in the packaging cells, the plan was to isolate individual clones by focus-purification under a semi-solid overlay, amplify them as outlined in Figure 2, and then perform nearly complete-length sequencing to identify the acquired mutations.

**Figure 3:**
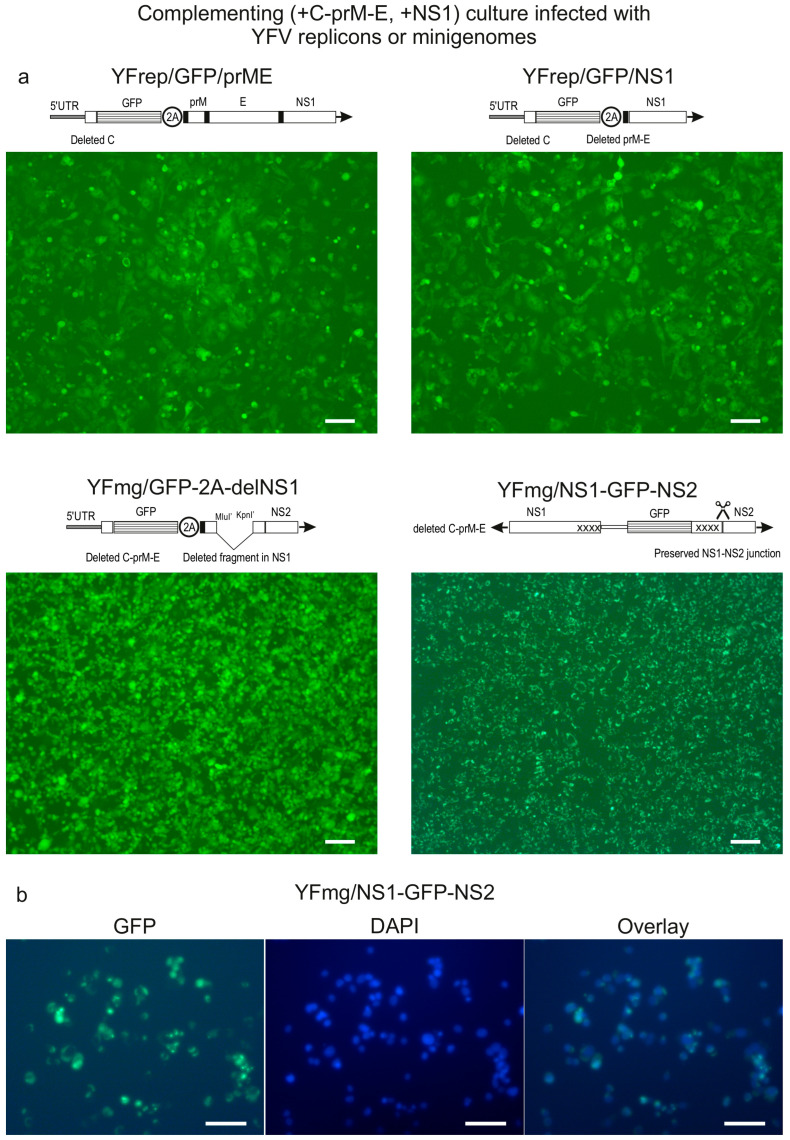
Transfection of YFV MICs into complementing cells. (**a**) Micrographs of GFP fluorescence in cell cultures at 72 h post-transfection with YFV MICs. A complementing cell line supplies, in trans, the NS1 protein necessary for the replication of minigenomes, as well as YFV structural proteins for packaging into infectious particles. Schematics of only the differing parts of the YFV RNAs are shown. The rightward and leftward arrows in the schematics indicate the continuation of the respective constructs. Scale bars, 100 µm. Objective magnification, 5×. (**b**) Micrographs of YFmg/NS1-GFP-NS2 in complementing cells, GFP fluorescence (**left**), DAPI (**center**) and overlay (**right**). Scale bars, 50 µm. Objective magnification, 20×.

The replication and packaging competence of the YFV-derived replicons and minigenomes was initially assessed by transfecting the complementing cell line, which supplies YFV NS1 and structural proteins. Upon transfection, all four variants propagated efficiently, with the proportion of GFP+ cells increasing over time. By 72 h, the majority of cells were GFP+ (Figure 3a). The observed efficient propagation was expected for the replicons. For the minigenomes, propagation in complementing cells demonstrates that the remaining replication machinery (NS2-NS5) efficiently utilizes NS1 supplied in trans. Infectious particle titers for YFmg/NS1-GFP-NS2 in these cells at 72 h post-transfection are presented in Table 1.

These findings indicate that modifications to NS1 (deletion or fusion) do not intrinsically preclude the packaging of replicating RNAs into infectious particles, provided that functional NS1 is available in the host cell.

Microscopic analysis revealed distinct fluorescence patterns of the GFP reporter. Variants designed for cytosolic GFP expression (YFrep/GFP/prME, YFrep/GFP/NS1 and YFmg/GFP-2A-delNS1) exhibited uniform, diffuse fluorescence throughout the cytoplasm (Figure 3a). In contrast, cells harboring the ‘NS1-GFP fusion’ minigenome display a different pattern (Figure 3b). Fluorescence was notably less intense, requiring longer exposure times for detection, and was concentrated in the perinuclear region. This localization is consistent with the expected endoplasmic reticulum (ER) lumen residency of the NS1-GFP protein. The dimmer fluorescence (of NS1-GFP compared to cytosolic GFP) could result either from the lower quantum yield of GFP in the ER lumen, from potential interference between the trans-supplied NS1 and the NS1-GFP or from less efficient folding; however, these possibilities were not investigated further.

The YFV replicons also replicated efficiently upon transfection of packaging cells (+C-prM-E, -NS1) and displayed fluorescence patterns indistinguishable from those observed in complementing cells at 72 h p.t. (comparable to the results shown for replicons in Figure 3). In contrast, both minigenomes transfected into the packaging cells yielded no GFP+ cells and produced no detectable infectious particles (Table 1). Even serial passaging of these cultures did not result in the emergence of fluorescence or signs of productive replication. Based on these results, the cultures were abandoned.

### 3.2. Adaptive Mutations from a ‘GFP-NS1’ Virus Partially Restore the Viability of the ‘NS1-GFP’ Minigenome

The failure to rescue YFmg/NS1-GFP-NS2—the initially created ‘NS1-GFP’ minigenome—which produced no infectious particles in packaging cells (Table 1), demonstrates that modifications to the C-terminus of NS1 can lead to non-viability, underscoring NS1’s critical role in viral replication. Nevertheless, we proceeded with efforts to engineer a replication-competent variant featuring the NS1-GFP fusion.

Our group previously generated a YFV-derived virus in which GFP is fused to the N-terminus of NS1 [42]. The initial variant in that study exhibited limited replication but subsequently acquired adaptive mutations, resulting in an efficiently replicating virus designated virGFP-NS1m6 [42]. These mutations include Q204R and A206V in GFP (positions renumbered to align with current standard GFP nomenclature), M108L in NS4A and a silent single-nucleotide change in NS4B, and P711A and K849Q in NS5.

Given that these substitutions function cooperatively for adaptation with the ‘GFP-NS1’ virus, we elected to test whether they could also restore viability to a variant with a different ‘NS1-GFP’ configuration. To this end, the substitutions were introduced into the YFmg/NS1-GFP-NS2 backbone in individual genes or in various combinations, generating the panel of variants described in Table 2.

All variants listed in Table 2 were transfected into packaging cultures, and then attempts were made to infect fresh monolayers of packaging cells with culture supernatants. Only two variants—YFVmg-v5/GFP* (NS4*) and YFVmg-v7/GFP* (NS4*, NS5*)—produced infectious particles at titers sufficient to infect new monolayers (Table 1). Upon transfection, these variants also yielded significantly higher proportions of GFP+ cells at 72 h (~30% and ~28%, respectively; gating strategy in Supplementary Figure S1; representative dot plots in Supplementary Figure S2).

**Figure 4:**
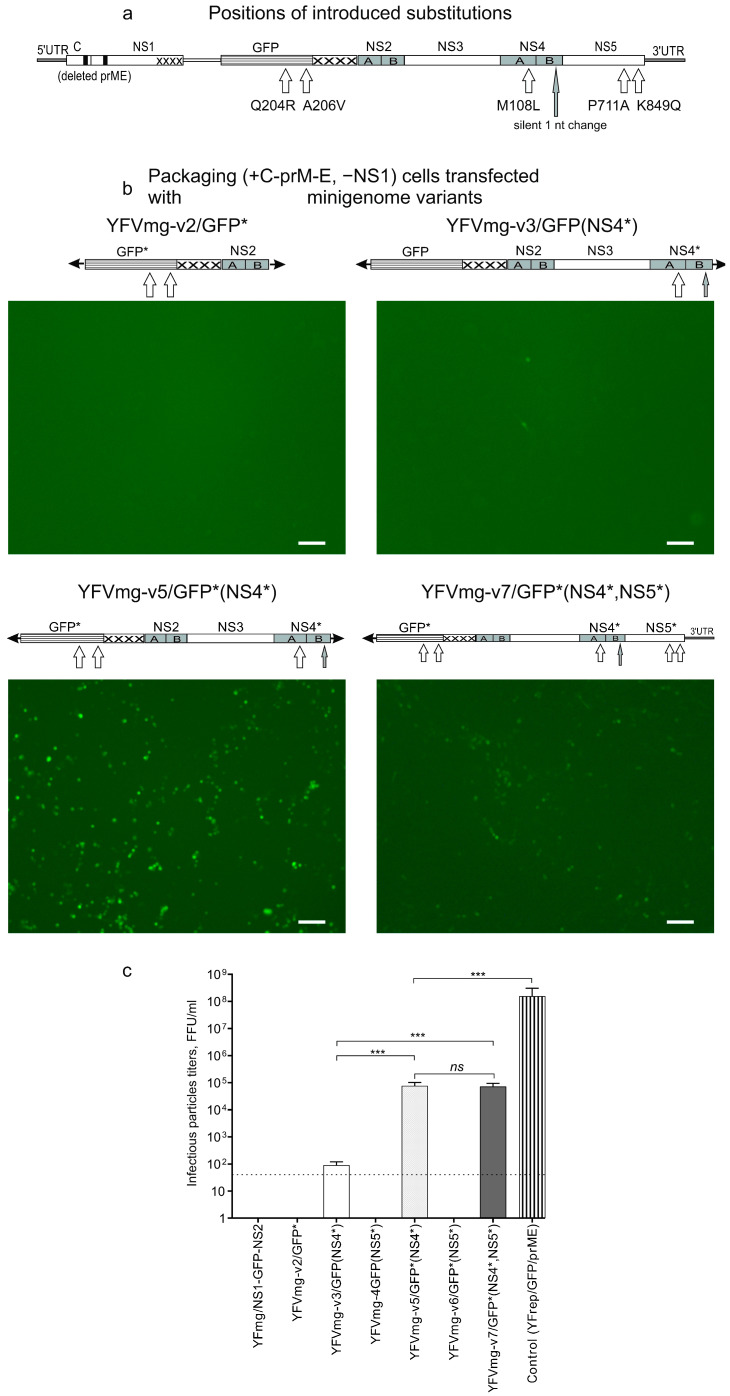
Positions in the ‘NS1-GFP’ minigenome variants harboring substitutions tested for adaptive effect and transfection results. (**a**) Scheme of the YFmg/NS1-GFP-NS2 minigenome, showing the positions of the planned substitutions. These substitutions originated from a culture-adapted virus containing a different fusion, ‘GFP-NS1’, as described previously [42]. In this study, previously found adaptive mutations were tested for their ability to restore viability in minigenomes having the ‘NS1-GFP’ fusion; (**b**) Fluorescence micrographs of packaging cells (+C-prM-E, −NS1) transfected with the indicated minigenomes, taken 72 h post-transfection. Above each micrograph is a schematic showing only the modified regions of the respective variant. Scale bars, 100 µm. Objective magnification, 5×; (**c**) Titers of infectious particles produced by the transfected cultures were measured as described in “Materials and Methods”. Titers are presented as mean ± SD, n = 3. The dashed line indicates the limit of detection. *** (*p* ≤ 0.001), ns (*p* > 0.05).

Micrographs of the transfected cultures are shown in Figure 4b. Only variants carrying both GFP and NS4A mutations (YFVmg-v5/GFP* (NS4*) and YFVmg-v7/GFP* (NS4*, NS5*)) produced detectable infectious particles (Table 1, Figure 4c). Their titers (~7.7 × 10^4^ and ~7.2 × 10^4^ FFU/mL, respectively) were sufficient to initiate infections in fresh cultures, yet remained three orders of magnitude lower than the control replicon YFrep/GFP/prME (Figure 4c), indicating that the NS1-GFP fusion only partially substitutes for native NS1. Notably, while NS5 substitutions enhance replication in the ‘GFP-NS1’ context [42], they provided no advantage here (cf. YFVmg-v5/GFP* (NS4*) vs. YFVmg-v7/GFP* (NS4*, NS5*) in Table 1).

### 3.3. Cell-Culture Adaptation in BHK-21 Cells Generated a Rearrangement Within the NS1-GFP Linker Region

**Figure 5:**
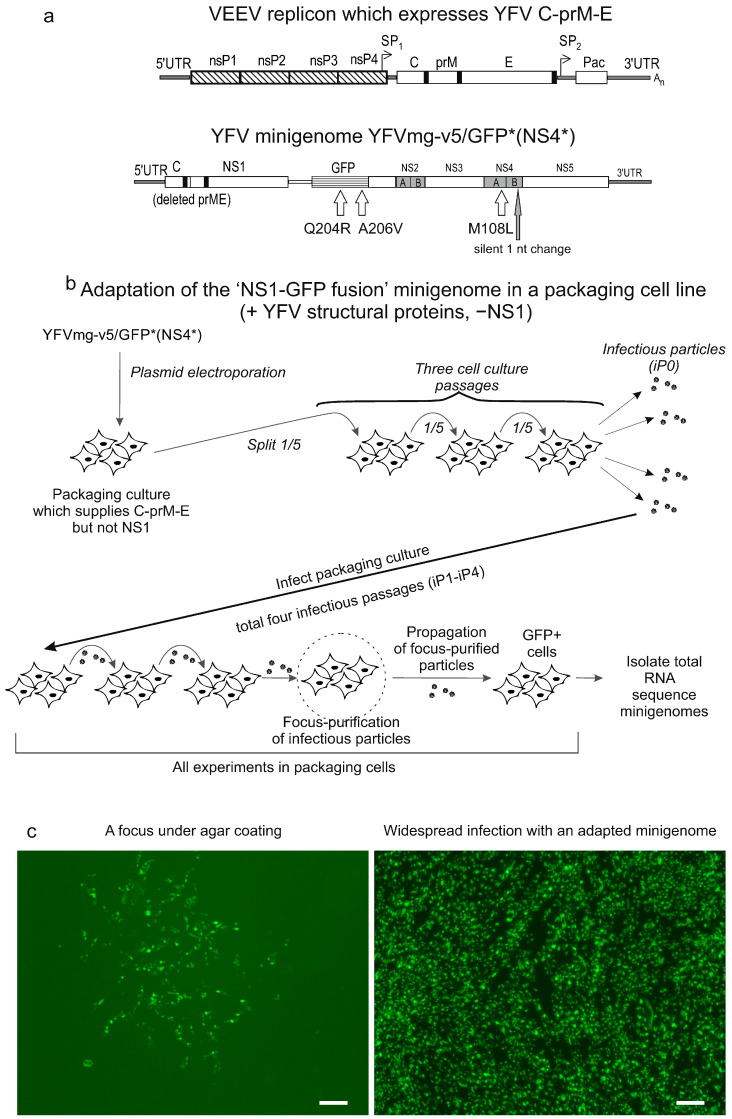
Adaptation of an NS1-GFP-fusion minigenome for replication in non-complementing BHK-21 cells. (**a**) VEEV replicon and YFV minigenome used in the adaptation experiment. Top: Schematic of the VEEV replicon (VEErep/C-prM-E/Pac), which supplies the YFV structural proteins C, prM and E. Bottom: Schematic of the variant YFVmg-v5/GFP* (NS4*), carrying introduced substitutions in GFP and NS4. (**b**) Experimental strategy: The YFVmg-v5/GFP* (NS4*) was transfected into packaging cells, which allow infectious propagation of YFV replicons. The initial transfected culture was serially passaged by cell splitting (passages P0–P3). Conditioned medium from the final split passage (P3) contained infectious particles and was used to initiate a series of infectious passages. Supernatant from each infectious passage was used to infect fresh packaging cells, enriching for adapted variants. (**c**) Phenotypic evidence of adaptation. Left panel: A representative fluorescent focus formed under an agar overlay at infectious passage 3 (iP3), demonstrating the emergence of efficiently spreading variants. Right panel: A culture of packaging cells infected with a focus-purified, adapted variant, showing widespread GFP expression indicative of efficient replication. Scale bars, 100 µm. Objective magnification, 5×.

Since the variant YFVmg-v5/GFP* (NS4*) showed slightly higher titers than YFVmg-v7/GFP* (NS4*, NS5*) (Figure 4c), it was selected for further work. Given the variant’s inherent replication capacity, we aimed to enhance its fitness through cell-culture adaptation. The experimental design is shown in Figure 5.

Packaging cells were electroporated with the YFVmg-v5/GFP* (NS4*) variant, and the culture was serially passaged as outlined in Figure 5. Conditioned medium collected from the passaged culture contained infectious particles and was used to initiate a series of infectious passages, with the first designated iP0 (Figure 5b). Adaptation of the variant was evident by infectious passage 3 (iP3), as it readily formed foci under an agar overlay (Figure 5c). Infectious particles eluted from three isolated foci were used to infect subconfluent monolayers of packaging cells, resulting in near-total infection (~100%) by 72 h post-infection (Figure 5c).

**Figure 6:**
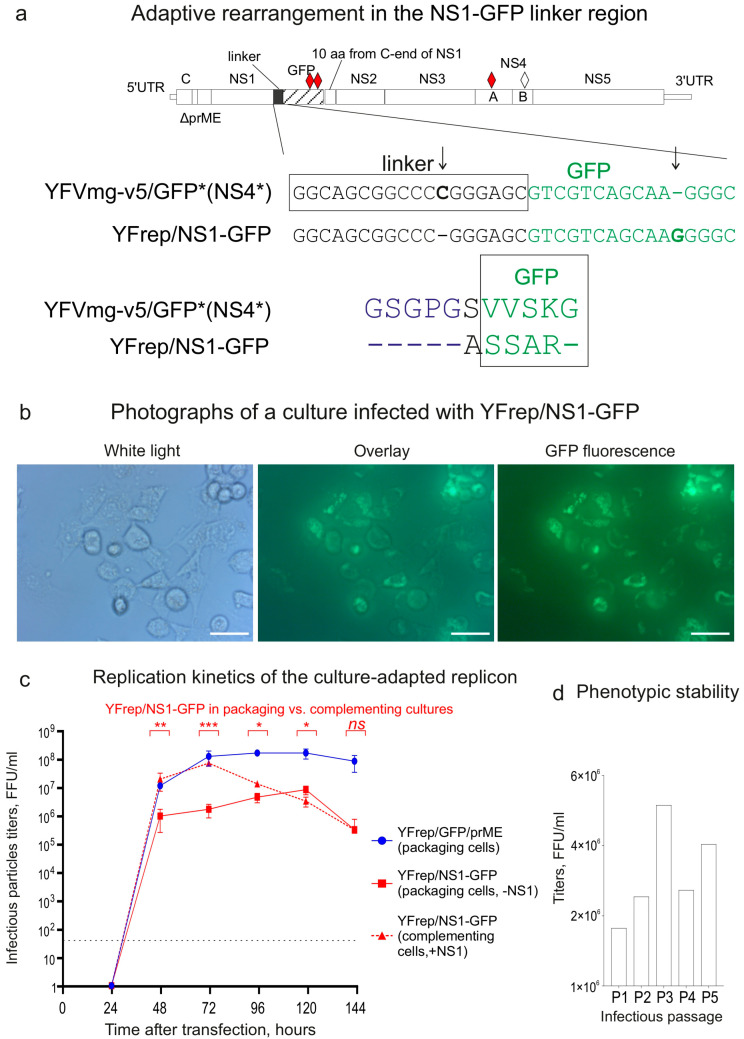
Adaptive changes in the NS1-GFP linker improve replication competence and result in a phenotypically stable replicon. (**a**) (**Top**): Schematic of the replicon encoding the NS1-GFP fusion protein. Red diamonds indicate positions of introduced co-adaptive substitutions. An open diamond marks the position of a silent nucleotide change. (**Bottom**): The culture-adapted replicon acquired rearrangements exclusively within the linker region between NS1 and GFP. Nucleotide and amino-acid sequence alignments show a single-C deletion (ΔC) and a G insertion (indicated by downward arrows in the figure), resulting in a 17-nt frameshift and an extended, less hydrophobic protein linker. The GFP reading frame begins with a valine codon rather than a methionine start codon, for cloning convenience. (**b**) Subcellular localization of the NS1-GFP protein by fluorescent microscopy. The panel shows GFP fluorescence (**right**), merged (**center**) and bright-field (**left**) images of cells infected with YFrep/NS1-GFP. Fluorescence is concentrated in the perinuclear region and in reticular structures consistent with endoplasmic reticulum localization. Scale bars, 20 µm. Objective magnification 40×. (**c**) Replication kinetics of YFrep/NS1-GFP in packaging (−NS1) and complementing (+NS1) cells. Infectious particle titers (FFU/mL) were measured over time after transfection. The control replicon YFrep/GFP/prME, expressing cytosolic GFP and containing the full YFV replication machinery, is shown for comparison. Data points represent mean ± SD from three biological replicates. The statistical significance of differences in means is shown for the titers of infectious particles of the adapted YFrep/NS1-GFP replicon in cultures of packaging (+C-prM-E, -NS1) and complementing (+C-prM-E, +NS1) cells. The dashed line indicates the limit of detection. ns (*p* > 0.05); * (*p* ≤ 0.05); ** (*p* ≤ 0.01); *** (*p* ≤ 0.001). (**d**) Phenotypic stability of YFrep/NS1-GFP. Infectious particle titers were measured at 72 h post-infection of packaging cells at a multiplicity of infection (MOI) of 0.1. Obtained titers (~2–5 × 10^6^ FFU/mL) are not markedly different from those produced following transfection, confirming the replicon’s phenotypic stability. Data represent a single experiment.

Full-genome sequencing of three focus-purified variants revealed an identical sequence featuring a rearrangement affecting the NS1-GFP linker region and the beginning of the GFP gene. This rearrangement involved a single cytosine deletion (ΔC) coupled with a guanine insertion (G) within oligo-homopolymeric tracts, resulting in a 17-nucleotide frameshift (Figure 6a). The frameshift altered the sequence of the linker and the GFP N-terminus, changing it from gsgpgSVVSKge to gsgpgASSARge (where altered residues are shown in uppercase). The new sequence is characterized by an extended tract of residues with small, less hydrophobic side chains. The key change involved replacing the valine-valine (VV) dipeptide with serine-serine (SS), which reduced side-chain volume and likely increased flexibility.

To confirm the adaptive role of this rearrangement, we performed reverse cloning of the modified NS1-GFP region into the YFVmg-v5/GFP* (NS4*) variant. The resulting adapted variant (replicon), designated YFrep/NS1-GFP, demonstrated stable replication in packaging cells independent of NS1 supplied in trans. Fluorescence microscopy revealed localization of fluorescence signal predominantly to the perinuclear region, as well as reticular distribution, consistent with ER-like localization of the NS1-GFP fusion protein (Figure 6b).

Assessment of replication kinetics was performed by transfecting packaging cells (+C-prM-E, −NS1) and complementing cells (+C-prM-E, +NS1). In packaging cells, the YFrep/NS1-GFP replicon exhibited slower replication kinetics and reached a peak titer approximately two orders of magnitude lower than that of the control replicon, YFrep/GFP/prME (Figure 6c). In complementing cells, however, the initial replication kinetics of YFrep/NS1-GFP were comparable to the control. This suggests that wild-type NS1 supplied in trans can compensate for the replication defect during the early stages of infection. However, at later time points (96–144 h), the titers in packaging and complementing cells equalized (Figure 6c). This convergence could be explained by several non-mutually exclusive mechanisms. First, the accumulating NS1-GFP fusion protein might eventually outcompete or interfere with the function of trans-supplied wild-type NS1, for instance by forming non-productive heterodimers or by saturating binding sites within the replication complex. Second, the initial high titers may decline due to the death of high-producer cells, leading to a late-stage culture enriched with cells in a physiological state that supports only limited production, with daily accumulation of infectious particles remaining below 10^5^ FFU/mL. We did not systematically monitor cell proliferation or viability in our experiments.

Phenotypic stability of the reconstructed replicon was assessed by performing five sequential infectious passages in packaging cells at a controlled multiplicity of infection (MOI) of 0.1. Titers harvested at 72 h post-infection ranged from 1.65 × 10^6^ to 5.15 × 10^6^ FFU/mL (Figure 6d), consistent with those produced by the YFrep/NS1-GFP replicon following transfection.

To rigorously evaluate the genetic stability of the YFrep/NS1-GFP replicon, RNA was isolated from pelleted infectious particles after the fifth passage. Sequencing of individual clones from the amplified NS1-GFP-NS2A region revealed no sequence heterogeneity among the clones, and their consensus sequence was identical to that of the input replicon. The threshold for detection of minor sequence variants was 10% (1/10), confirming the high genetic stability of the adapted YFrep/NS1-GFP replicon.

### 3.4. A Replication-Competent YFV Replicon Expresses a Stable NS1-GFP Fusion Protein

**Figure 7:**
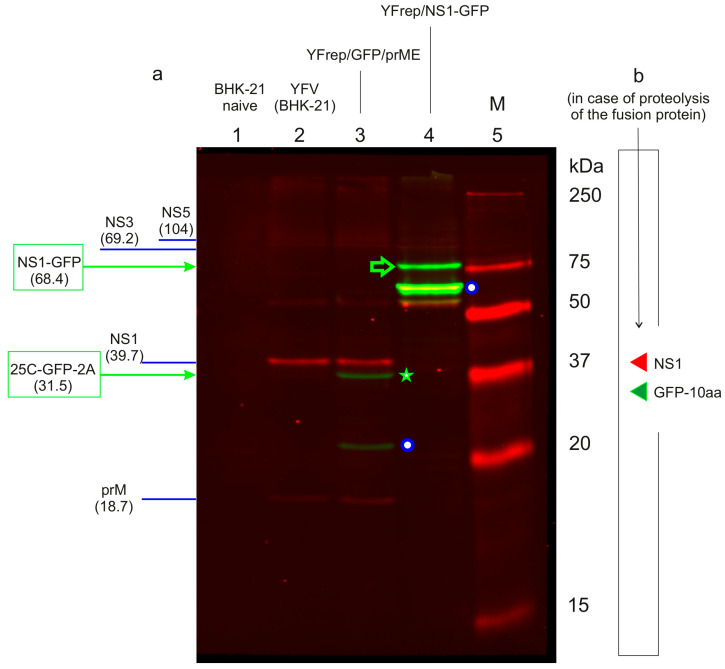
Western blot analysis of intracellular proteins in cells harboring YFV or YFV-derived replicons. (**a**) The photograph shows a membrane from a two-color immunoblot of cell lysates stained with a polyclonal mouse anti-YFV antiserum (red channel, 700 nm) and an anti-GFP antibody (green channel, 800 nm). Imaging was performed on a LI-COR Odyssey system. Lane designations: 1, Naive BHK-21 cells (uninfected control); 2, BHK-21 cells infected with wild-type YFV; 3, Packaging cells harboring the YFrep/GFP/prME replicon, expressing the 25C-GFP-2A fusion protein; 4, Packaging cells harboring the YFrep/NS1-GFP replicon, expressing the NS1-GFP fusion protein; 5, Protein marker (Precision Plus Protein Kaleidoscope, Bio-Rad #1610375); only marker bands detected in the 700 nm channel are visible. Molecular weights (in kDa) of the protein marker are indicated on the right. Key findings: YFV-specific proteins (red bands) detected in lanes 2–4: NS5 (104 kDa), NS3 (69.2 kDa), NS1 (39.7 kDa) and prM (18.7 kDa). GFP-containing fusion proteins (green bands) were detected in lanes 3 and 4. Lane 3: The band corresponding to the 25C-GFP-2A fusion protein (~31.5 kDa) is indicated by an asterisk (★). Lane 4: The band corresponding to the full-length NS1-GFP fusion protein (~68.4 kDa) is indicated by a double green arrow (⇒). In lanes 3 and 4, circles (◯) mark unidentified GFP-containing bands at approximately 20 kDa and 60 kDa, respectively, likely representing degradation or proteolytic fragments. (**b**) Positions of potential proteolytic cleavage products should the NS1-GFP fusion protein (from the YFrep/NS1-GFP replicon) be cleaved, releasing full-length NS1 (39.7 kDa, red triangle) and the C-terminal fragment (marked ‘GFP-10aa’, 28.7 kDa, green triangle).

To confirm that the YFrep/NS1-GFP replicon successfully produces the designed fusion protein, we performed Western blot analysis on lysates from infected cells (Figure 7; a scan of the entire membrane is provided in Supplementary Figure S3). In cells harboring the YFrep/NS1-GFP replicon, two-color immunoblotting with anti-YFV and anti-GFP antibodies revealed a prominent band of approximately 68 kDa, consistent with the expected size of the full-length NS1-GFP fusion protein (68.4 kDa) (Figure 7, lane 4). The same band was detected by both antibodies, confirming its identity as a chimeric protein containing both NS1 and GFP epitopes. This specific band was absent in control lysates from naive BHK-21 cells (lane 1), cells infected with wild-type YFV (lane 2) and cells harboring the YFrep/GFP/prME replicon (lane 3), which contains the wild-type YFV replication machinery.

As expected, the native NS1 protein (39.7 kDa) was detected by the anti-YFV antibody only in lysates from wild-type YFV-infected cells (lane 2) and in YFrep/GFP/prME-harboring cells (lane 3). No band corresponding to wild-type NS1 (~40 kDa) is visible in lane 3. This confirms that the replication capacity of the YFrep/GFP/prME replicon does not arise from proteolytic cleavage that would release NS1 from the fusion protein.

The YFrep/GFP/prME replicon expresses a cytosolic 25C-GFP-2A fusion protein, which was specifically detected by the anti-GFP antibody as a 31.5 kDa band (lane 3).

Smaller GFP-reactive bands (~60 kDa in lanes 3 and ~20 kDa in lane 2) likely represent proteolytic fragments of the respective fusion proteins. Importantly, the blot also confirmed other key viral non-structural proteins, NS5 (104 kDa) and NS3 (69.2 kDa), in cells harboring YFV-derived RNAs.

A key finding of this immunoblotting experiment is the confirmation that the engineered YFrep/NS1-GFP replicon expresses the NS1-GFP fusion protein and that replication most likely utilizes this fusion protein. This finding provides a molecular tool for both the biochemical study of GFP-tagged replicative complexes and further modification of the replicon to enable proximal labeling of the unknown protease responsible for cleaving the NS1-NS2 junction.

### 3.5. The NS1-GFP Fusion Protein Localizes to a Detergent-Resistant Heavy Membrane Fraction

**Figure 8:**
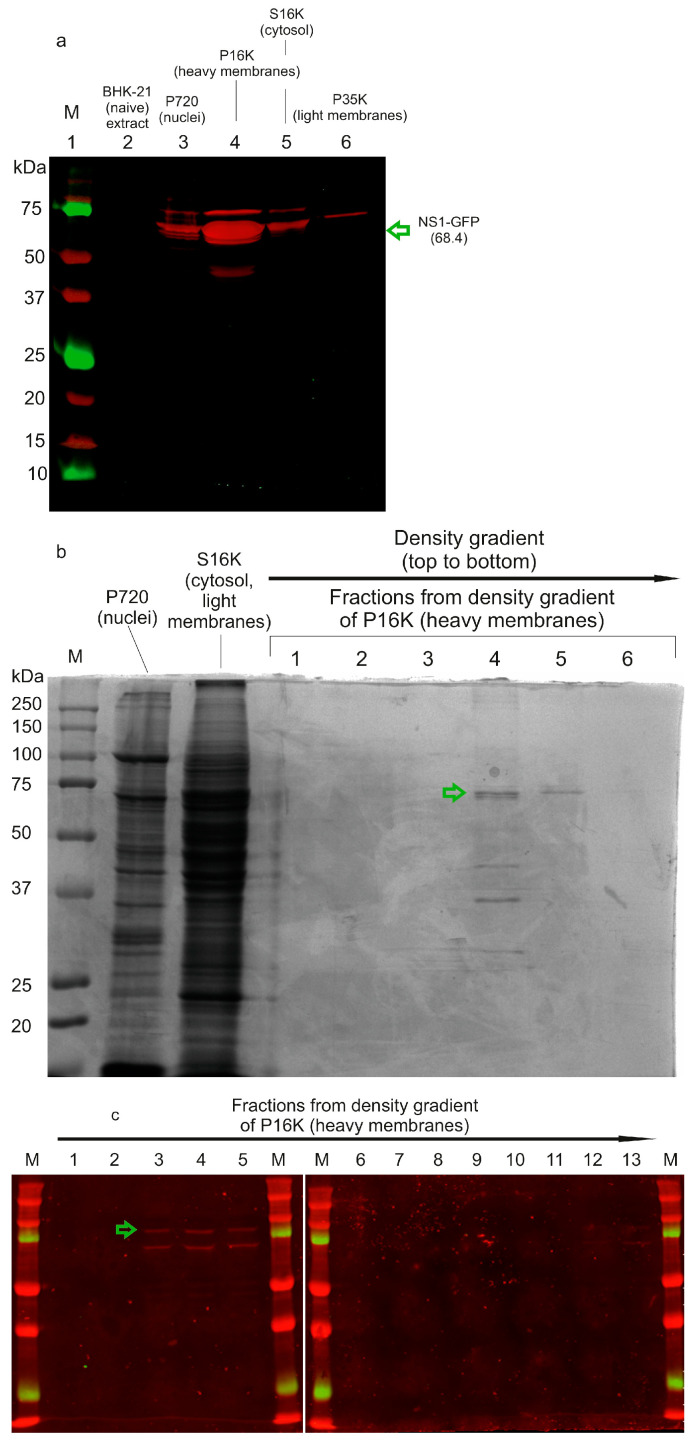
Subcellular localization of the NS1-GFP fusion protein determined by biochemical fractionation and flotation gradient centrifugation. (**a**) Western blot analysis of fractions from BHK-21 cells harboring the YF replicon encoding NS1-GFP (lanes 3–6), probed with an anti-NS1 antibody. Lane 1, Precision Plus Protein Kaleidoscope marker (Bio-Rad Cat. 1610375); 2, whole-cell lysate of naïve BHK-21 cells (uninfected control); 3, nuclei (P720, pellet at 720× *g*); 4, heavy membranes (P16K, pellet at 16,000× *g*); 5, post-mitochondrial fraction (S16K, supernatant after 16,000× *g*); 6, light membranes (P35K, pellet after ultracentrifugation (35,000× *g*) of the S16K fraction). This blot demonstrates that NS1-GFP predominantly partitions into the heavy membrane fraction (P16K). (**b**) Coomassie-stained SDS-PAGE gel of the heavy membrane (P16K) fraction subjected to flotation centrifugation. The heavy membrane (P16K) fraction was separated by flotation centrifugation in a sucrose gradient. Samples were analyzed by SDS-PAGE and Coomassie staining. Lane M, Marker, same as in panel (**a**); P720, nuclei; S16K, cytosol; Gradient fractions 1–6 upon separation of heavy membranes. The band indicated by the green arrow (~70 kDa) was excised and analyzed by mass spectrometry, confirming the presence of the NS1-GFP fusion protein (Supplementary Figure S7). (**c**) Western blot analysis of gradient fractions from an experiment similar to that shown in panel (**b**). Proteins were probed with an anti-NS1 monoclonal antibody. Lanes: M, marker (as in panel (**a**)); 1–13, gradient fractions. Double green arrows (**a**–**c**) indicate the NS1-GFP band (68.4 kDa). The results (**b**,**c**) are consistent with the interpretation that NS1-GFP marks the presence of YFV replication complexes (RCs), which are associated with detergent-resistant heavy membranes.

Using an NS1-GFP-encoding replicon, we confirmed the localization of the fusion protein to intracellular heavy membranes—the established site of flavivirus replication complexes. BHK-21 cells (~10^8^ cells) were transfected with YFrep/NS1-GFP. At 48 h post-transfection, cells were lysed in 1% Triton X-100 and subjected to biochemical fractionation as described in Materials and Methods (Section 2.13, Isolation of Heavy Membranes). Western blot analysis of the subcellular fractions revealed that NS1-GFP was predominantly detected in the heavy membrane fraction (P16K), with minimal signal in the nuclear (P720), post-mitochondrial (S16K), or light membrane (P35K) fractions (Figure 8a; a complete membrane scan is provided in Supplementary Figure S4).

The biochemical properties of NS1-GFP were further investigated by flotation gradient centrifugation of the P16K fraction. NS1-GFP floated upward through the sucrose gradient, accumulating in fractions above the 55% sucrose layer—consistent with its association with protein-rich detergent-resistant heavy membranes (Figure 8b,c; complete images are provided in Supplementary Figures S5 and S6). Importantly, the dominant band (Figure 8b) was excised and subjected to mass spectrometry, confirming the presence of the NS1-GFP fusion protein (Supplementary Figure S7).

One conclusion from this experiment is that the NS1-GFP fusion protein is produced at high levels in infected cells, enabling its direct detection by Coomassie staining on SDS-PAGE gels from lysates of ~10^8^ cells. The biochemical fractionation and flotation assays collectively demonstrate that the NS1-GFP fusion protein specifically enriches in the detergent-resistant heavy membrane compartment, a hallmark of the flavivirus replication organelle (RO).

## 4. Discussion

Flaviviruses include pathogens of significant medical importance, making a detailed understanding of their replication and pathogenicity mechanisms crucial for developing specific antiviral inhibitors. A significant contribution may come from studies that use reverse genetics to make targeted modifications to the viral replication machinery, thereby elucidating its underlying mechanisms. The flavivirus genome imposes constraints on the insertion of extended foreign sequences, a challenge that is evident within the nonstructural (NS) protein region. While the structural protein region can be extensively modified to create replicons or chimeric viruses, successfully generating genetic fusions of heterologous functional proteins to flavivirus NS proteins while maintaining virus viability has proven far more challenging.

For example, Eyre et al. [43] assessed genetic flexibility in the dengue virus genome through random transposon-mediated insertions of 15-nt (5-a.a.) linkers at various positions. They identified numerous sites that tolerate these short insertions without disrupting viral replication; yet only one location, a highly flexible region within the NS1 protein immediately downstream of Lys-174, proved permissive for the insertion of full-length heterologous functional proteins such as APEX2, Nanoluc and the fluorescent marker mScarlet, thereby enabling the generation of infectious, reporter-tagged viruses.

The study by Syzdykova et al. [42] reports the generation of a replication-competent yellow fever virus (YFV) variant in which GFP is fused to the N-terminus of NS1 to produce a GFP-NS1 fusion protein. While the initially rescued GFP-NS1 virus replicated poorly, serial passaging in cell culture selected for adaptive mutations that restored efficient replication and resulted in a genetically stable virus. These adaptive mutations were located in GFP, NS4A, NS4B and NS5. The study demonstrates that a large GFP tag can be genetically fused to the small and functionally critical N-terminal β-roll domain of NS1 and that the resulting GFP-NS1 fusion protein functions in place of native NS1 to support viral replication.

In their work, Ma et al. [39] constructed a replication-competent Japanese encephalitis virus (JEV) with a hemagglutinin (HA) tag fused to the N-terminus of NS2A. The resulting virus stably maintained the genetic modifications through serial passages, enabling the use of anti-HA antibodies to study NS2A localization.

Tamura et al. [45] inserted the 11-a.a. HiBiT subunit of NanoLuc luciferase into the N-terminus of NS1 in the JEV genome. To preserve the E/NS1 junction, four a.a. residues from the N-terminus of NS1 were duplicated. The resulting virus produced titers comparable to the parental strain and maintained the HiBiT insert over five passages. In the subsequent work [46], the same group placed the HiBiT tag into NS1 after residue 349, resulting in a stable reporter virus that maintained near-wild-type virulence in mice.

Rumyantsev et al. [47] employed transposon-mediated random insertions to introduce the small M2e epitope peptide (35 a.a.) from influenza A virus into the NS1 protein of the chimeric flavivirus ChimeriVax-JE. They created a library of random insertion mutants across the NS1 gene. From this heterogeneous population, they isolated a highly replication-competent viral clone containing the foreign M2e epitope after residue 236. The insertion impaired the ability of the NS1 protein to dimerize. However, this insertion was genetically stable over 12 serial passages.

Collectively, the presented studies demonstrate that specific sites within NS1 tolerate foreign insertions without a significant loss of viral viability. In contrast, the NS2A protein has been shown to be nearly non-permissive to insertions, even of small sequences [43]. Unlike these insertions within the NS1 body, our study engineered a replicon with a large functional GFP tag positioned just ten a.a. upstream of the cleavage site (Table 3). Introducing a genetically encoded functional tag in this location may have implications for the future identification of the protease responsible for cleaving the NS1-NS2A junction via proximity-dependent labeling or cross-linking strategies. The use of replicons and minigenomes in place of an infectious virus significantly reduced the biocontainment requirements, enabling this research to be performed at BSL-2. This methodological shift from our previous work [42] was mandated by updated national regulations.

To enable cell-to-cell spread of YFV replicons in culture, we developed cell lines persistently infected with a non-cytopathic Venezuelan equine encephalitis virus (VEEV) replicon, which produces structural proteins C-prM-E. This helper VEEV replicon constitutively expresses YFV proteins and a puromycin resistance marker, allowing for the selection and long-term maintenance of BHK-21 cell lines stably expressing the required YFV proteins.

Furthermore, the initial minigenome (YFmg/NS1-GFP-NS2, Figure 1) was non-viable in naive cells, necessitating the development of a trans-complementation system to supply NS1 in trans. For this purpose, an additional helper VEEV replicon was engineered to express the YFV proteins C-prM-E-NS1.

The initial minigenome YFmg/NS1-GFP-NS2 could be trans-complemented in NS1-producing cells, where it produced high titers of infectious particles (Table 1). However, it remained non-viable in packaging cells lacking NS1 expression and yielded no detectable infectious particles (Table 1). Viability was partially restored through the introduction of a combination of adaptive mutations derived from a previously described GFP-tagged virus with a different insertion topology [42]. Cloning the described substitutions in various combinations established that the required set for rescue comprised substitutions in GFP (Q204R/A206V) and in NS4A (M108L). The replicon RNA bearing the identified substitutions replicated in cells without NS1 complementation and produced GFP with a fluorescence pattern consistent with endoplasmic reticulum (ER) localization. However, its slow propagation in culture prompted an adaptation experiment to select a more robust variant (Figure 5). This experiment yielded a variant capable of forming foci under agar overlay and infecting the majority of cells. Sequencing revealed a 17-nt frameshift within the NS1-GFP linker that encoded a series of non-conservative substitutions. These changes reduced side chain volume, resulting in a longer and less hydrophobic linker between NS1 and GFP. A replicon engineered to incorporate this modification (YFrep/NS1-GFP) replicates autonomously in naive BHK-21 cells. In the packaging cell culture (complementing C-prM-E), the replicon YFrep/NS1-GFP produces titers of ~10^6^ FFU/mL and is genetically stable over multiple passages.

Western blot analysis (Figure 7a) confirmed the expression of an NS1-GFP fusion protein of the expected size (68.4 kDa). Considering the detection limit of the method (~1 ng of GFP, determined empirically as described in Materials and Methods Section 2.12) and the positions of the projected cleavage products marked in Figure 7b, we conclude that the intensity of any potential band corresponding to mature NS1 (39.7 kDa) or the GFP-containing fragment (28.7 kDa) is at least two orders of magnitude lower than the intensity of the full-length NS1-GFP band. Therefore, the chimeric protein does not undergo significant proteolytic cleavage in cells. Fluorescence microscopy revealed a characteristic perinuclear/reticular pattern consistent with endoplasmic reticulum localization of the fusion protein. Finally, biochemical isolation of a detergent-resistant heavy membrane fraction from cells infected with the replicon, followed by mass spectrometry and Western blot analysis, confirmed that the NS1-GFP fusion protein specifically co-fractionated with this compartment (Figure 8). This association, validated by both differential centrifugation and flotation gradient assays, is a hallmark biochemical property of proteins residing within the flavivirus replication organelle.

The adapted replicon YFrep/NS1-GFP produced titers of approximately 10^6^ FFU/mL in the packaging cell line (+C-prM-E, -NS1). Initial titers in the complementing cell line (+C-prM-E, +NS1) were significantly higher, reaching up to 10^8^ FFU/mL (*p* ≤ 0.01). This difference persisted until 96 h post-transfection (Figure 6c), indicating that trans-complementation by wild-type NS1 is stage-dependent and that the NS1-GFP fusion provides only partial functional substitution for native NS1. The persisting deficiency in titers is most consistent with impaired RNA replication and/or inefficient packaging of RNA, and/or virion formation. Nevertheless, our results demonstrate that the extreme C-terminus of NS1 can tolerate the insertion of large foreign sequences.

The higher initial titer in complementing cells likely reflects a transient boost because of the trans-supplied wild-type NS1 accumulated in cells, which appears to efficiently support early replication and packaging of YFV variants with modified (suboptimal) NS1. At later time points, however, the titers in packaging and complementing cultures converged (Figure 6c). As the infection progresses, the accumulating NS1-GFP fusion protein may interfere with the function of trans-supplied wild-type NS1, potentially through the formation of non-productive heterodimers or competition for binding sites within the replication complex. Alternatively, the titer decline could result from culture deterioration over prolonged incubation, including loss of high-producing cells and the onset of a low-production physiological state in confluent monolayers.

Our experiments demonstrated that autonomous replication of our NS1-GFP variant was achieved only after the introduction of co-adaptive mutations in both the NS1-GFP fusion and NS4A, suggesting synergy. This aligns with the established functional interdependence between NS1 and transmembrane proteins NS4A/NS4B in the flavivirus replication complex. NS4A and NS4B are neighboring proteins in the polyprotein separated by a short hydrophobic peptide known as “2k”, which acts as a signal sequence for NS4B translocation into the ER lumen. Both NS4A and NS4B are hydrophobic transmembrane proteins with multiple transmembrane domains [18,19].

The functional interaction between NS1 and NS4A was first established in the seminal work by Lindenbach and Rice, which demonstrated that YFV could not utilize dengue virus NS1 without a compensatory mutation (N42Y) in NS4A [33]. Similarly, a West Nile virus NS1 mutant (RQ10NK) is rescued by a suppressor mutation in NS4B (F86C) [48]. The functional importance of these interactions was underscored by biochemical studies in a yeast two-hybrid screen mapping the binding interface between dengue virus NS1 domain II and the cytoplasmic tail of NS4A [49]. Disruption of this interface by the NS4A-Y41F mutation abrogates binding and impairs viral fitness [49]. Collectively, these works establish that the NS1-NS4A/NS4B interfaces are crucially essential for replication. Our results, that efficient replication of the NS1-GFP replicon requires the NS4A-M108L substitution, align with these findings. However, to date, we cannot produce a mechanistic model for the role of NS4A-M108L, as NS4A is a small protein dominated by transmembrane segments and M108 maps to the middle of a predicted transmembrane domain. The role of the silent adenine-to-guanine mutation at nucleotide position 213 of the NS4B gene was not investigated separately.

The co-adaptive GFP mutations (Q204R and A206V) present an intriguing finding that requires mechanistic investigation. Position A206 is a well-established site controlling the dimerization of *Aequorea victoria* GFP-derived fluorescent proteins. The canonical A206K mutation introduces a charged lysine residue that prevents GFP dimerization and is widely used to generate monomeric variants [50,51]. In contrast, our adapted replicon selected for a hydrophobic valine residue (A206V) at this position. Similarly, glutamine 204 (Q204) is located on the surface of the β-barrel within a known dimer interface, and its substitution for histidine (Q204H) has been reported to promote dimerization [52]. The Q204R mutation in our construct introduces a stronger positive charge at this same interface. These observations lead to a testable hypothesis: the Q204R/A206V combination may alter the oligomerization propensity of the GFP moiety, potentially promoting dimerization, which could in turn facilitate or stabilize the essential dimeric state of the NS1 moiety within the fusion protein. This would align with the critical role of NS1 dimerization in membrane remodeling and replication organelle biogenesis [13]. However, alternative explanations are possible, such as the mutated residues in GFP could influence the folding efficiency, thermodynamic stability, or ER maturation kinetics of the GFP tag. Altered surface properties of the GFP variant might modulate its interaction with other components of the replication machinery independently of any effect on NS1 dimerization. Improved folding or stability of GFP might indirectly aid the correct folding or ER retention of the entire NS1-GFP fusion.

The fact that the same GFP mutations (Q204R/A206V) were independently selected to restore fitness in a YFV with an N-terminal GFP-NS1 fusion [42] suggests their effect is not specific to the C-terminal configuration but reflects a general, position-independent influence on the chimeric protein’s function. In this regard, the dimerization hypothesis provides a unifying model that could explain the benefit of these mutations in both topological contexts. We postulate that fusing a large tag—monomeric GFP—to NS1, either at its N- or C-terminus, disrupts the natural ability of NS1 to dimerize. In its native state, NS1 rapidly forms dimers, and structural studies confirm that the dimeric form (dNS1) possesses key interfaces for membrane interaction and endoplasmic reticulum remodeling, essential for replication organelle (RO) biogenesis [13]. The loss of dNS1 formation would impair these critical functions, preventing proper RO assembly and rendering the virus non-viable. Therefore, the Q204R/A206V mutations in GFP may restore viral replication by promoting GFP dimerization, which in turn could facilitate or stabilize the correct dimeric conformation of the NS1 moiety within the fusion protein. This model explains why the same mutations are beneficial in both fusion orientations and underscores the critical importance of the NS1 dimeric state for the formation of a functional replication organelle. However, direct experimental evidence is required. Future work should explicitly test the oligomeric state of the NS1-GFP fusion protein carrying wild-type versus Q204R/A206V GFP and probe its interactions with partners like NS4A.

A frameshift rearrangement within the NS1-GFP fusion changed five a.a. residues at the very beginning of GFP (Figure 6), and this rearrangement was selected for replicative fitness. Notably, the very C-terminal residues of NS1 are not resolved in X-ray crystallographic structures (PDB: 4O6B, 4O6C, 4O6D), which is consistent with the high conformational flexibility of the C-terminus, and seems at odds with the sequence-selection found in our study. We postulate that while the NS1 C-terminus may be flexible in biochemically isolated NS1, within the functional replication complex, its conformation is likely constrained by interactions with other viral components (e.g., NS2A, NS4A, or membranes). The adaptive linker rearrangement may therefore stabilize a specific, functionally required conformation. The use of a longer linker sequence may further improve replication efficiency, although this possibility was not explored in the present study.

Importantly, the resulting NS1-GFP replicon demonstrated high genetic and phenotypic stability over five serial passages in packaging cells. This stability was confirmed not only by consistent infectious titers (Figure 6d) but also by sequence analysis of the passage 5 infectious particles, which showed no sequence heterogeneity in the NS1-GFP-NS2A region and matched the sequence of the transfected replicon. For large-scale production, the NS1-GFP replicon titers can be substantially increased (to approximately 10^8^ FFU/mL) by passaging the replicon in complementing cells (+C-prM-E, +NS1). This has practical significance, as we currently employ infection of naïve BHK-21 cells with packaged replicon particles—rather than transfection with the molecular clone—to generate the heavy membrane fractions used for biochemical studies of the replication complexes.

The emergence of an adaptive frameshift raises questions about its origin. While the YFV 17D vaccine strain exhibits high genetic stability in vivo, this apparent conservatism may reflect stringent selection rather than inherent polymerase accuracy. The frameshift mutation resulting from a combined deletion and insertion within homopolymeric tracts points to a slippage-mediated frameshift mutation mechanism. While selection pressure is the obvious driver for the fixation of the fittest variant, we also hypothesize that the mutation rate may be intrinsically higher in genomes carrying large foreign inserts. More specifically, we postulate that the native genomic RNA is structured in a way that minimizes replication errors. If this is the case, the GFP insertion could disrupt these stabilizing structures, creating a local mutational hotspot and increasing the error rate at this specific site. Future studies that correlate changes in local RNA structure with mutation rates could shed further light on this possibility.

The results of this study naturally lend themselves to integration with ultrastructural approaches. First, the NS1-GFP-fusion replicon provides a platform for ultrastructural analysis of the flavivirus replication organelle (RO) using correlative light and electron microscopy (CLEM) and electron tomography. The GFP tag allows for live-cell fluorescence imaging to identify infected cells and specific RO-rich regions of interest, which can then be precisely targeted for high-resolution electron microscopy (EM) or focused ion beam scanning electron microscopy (FIB-SEM), to precisely map the sub-ER compartment where the intact fusion protein accumulates, which should coincide with the site of the cleavage of interest. Second, the described genetic design can be modified to express an NS1-TurboID-NS1′ fusion, where the promiscuous biotin ligase TurboID replaces GFP. Upon activation, TurboID would biotinylate proximal proteins, including the elusive protease, at the cleavage site. The biotinylated proteome from isolated heavy membranes (as shown in Figure 8) could then be captured on streptavidin beads and analyzed by mass spectrometry. Candidate proteases identified via this proximity proteomics screen could be validated functionally by siRNA knockdown or CRISPR knockout in the replicon system, assessing cleavage efficiency via Western blot for the NS1-GFP fusion or a complementary reporter assay. Cryo-electron tomography (cryo-ET) studies could reveal the native architecture of the replication complex containing the NS1-GFP fusion and additional tags, depending on the chosen experimental strategy. Such a combination of spatial information from ultrastructural studies and molecular identity from proximity labeling could result in a plausible hypothesis about the protease’s identity (e.g., an intramembrane protease or a lumenal protease with restricted access, etc.).

It is possible to propose several readily achievable applications for the NS1-GFP fusion in studies of the replication organelle (RO). The genetic stability of the replicon allows for the generation of sufficient material for purification to a high degree of purity. Furthermore, the stability of the GFP-bearing replicon suggests that the same genetic design can be adapted to create fusions with more complex, multi-component tags. We plan to modify GFP by adding an affinity tag suitable for affinity chromatography. In this case, it would become possible to purify the RO using a combination of ultracentrifugation and affinity chromatography, which should theoretically yield a purity of isolated ROs that has not yet been described in the literature.

The YFrep/NS1-GFP replicon is one of the few engineered flaviviruses available to date in which a replication-complex protein is genetically fused to a fluorescent protein. As demonstrated, the NS1-GFP fusion enables real-time visualization of the viral replication machinery in cells, as well as biochemical isolation and subcellular fractionation of its GFP-labeled components (Figure 8). A natural extension of this system is the incorporation of a proximity-labeling enzyme, such as TurboID, either in place of GFP or fused to its C-terminus, to label and identify proximal interactors. Our replicon positions the heterologous insertion just 10 a.a. upstream of the cleavage site, significantly closer than any previously described permissive site for a large functional tag. This unique proximity makes the system ideally suited for capturing the still-unknown NS1-NS2A protease via proximity-labeling strategies.

Finally, the described replicon-based system, rather than a full infectious virus, significantly lowers the biosafety barrier for studying this critical region of the flavivirus genome. By operating under BSL-2 containment, this platform makes future research, including the use of NS1-GFP or NS1-TurboID derivatives to identify the NS1-NS2A protease, accessible to a wide range of academic laboratories that lack access to the BSL-3 facilities typically required for working with replicable YFV.

## 5. Conclusions

We describe a replication-competent yellow fever virus replicon featuring a GFP tag fused to the NS1 C-terminus and positioned 10 amino acids upstream of the NS1-NS2A cleavage junction. Through the accumulation of adaptive mutations, this replicon is capable of replicating in naive cells without NS1 trans-complementation. This platform can be further modified to incorporate additional functional genes near the NS1-NS2A cleavage site and adapted for studying the still-unknown mechanism of NS1-NS2A processing using proximity labeling or crosslinking approaches.

## Figures and Tables

**Table 1:** Infectious particle titers of YFV-derived constructs in complementing and packaging cell lines.

Variant	Wild-Type NS1 Supplied in Trans, Yes or No	Titer (FFU/mL) ^1^
YFmg/NS1-GFP-NS2	Yes	(4.08 ± 2.24) × 10^7^
YFmg/NS1-GFP-NS2	No	<LOD
YFVmg-v3/GFP (NS4*)	No	(9.13 ± 2.30) × 10^1^
YFVmg-v5/GFP* (NS4*)	No	(7.67 ± 2.52) × 10^4^
YFVmg-v7/GFP* (NS4*, NS5*)	No	(7.17 ± 2.36) × 10^4^
YFrep/NS1-GFP	No	(2.02 ± 1.03) × 10^6^
YFrep/NS1-GFP	Yes	(9.11 ± 1.38) × 10^7^
YFrep/GFP/prME (control replicon)	Endogenous wild-type NS1	(1.59 ± 0.87) ×10^8^

Notes: ^1^ Infectious particle titers are shown in samples of the culture medium collected at 72 h post transfection. The titers are shown as mean ± SD from three independent transfections. Limit of detection (LOD) is 50 FFU/mL.

**Table 2:** Replication efficiency of ‘NS1-GFP’-type minigenome variants containing mutations in GFP, NS4 and NS5.

Minigenome	Location of Mutations (Introduced Amino Acid Substitutions) ^1^	Qualitative Description by Microscopy ^2^	Percentage of GFP+ Cells by Flow Cytometry ^3^
Initially developed minigenome			
YFmg/NS1-GFP-NS2	Wild-type eGFP and YFV sequences	No GFP+ cells	<0.01%
Introduced changes			
YFVmg-v2/GFP*	GFP (Q204R, A206V)	No GFP+ cells	<0.01%
YFVmg-v3/GFP(NS4*)	NS4 (M108L and a silent nt change)	<1 GFP+ cell per field	<0.5%
YFVmg-4GFP(NS5*)	NS5 (P711A, K849Q)	No GFP+ cells	<0.01%
YFVmg-v5/GFP* (NS4*)	GFP (Q204R, A206V), NS4 (M108L and a silent nt change), NS4 (M108L)	Visible foci of GFP+ cells	30.4% (20–39%) ^4^
YFVmg-v6/GFP*(NS5*)	GFP (Q204R, A206V), NS5 (P711A, K849Q)	No GFP+ cells	<0.01%
YFVmg-v7/GFP*(NS4*, NS5*)	GFP (Q204R, A206V), NS4 (M108L and a silent nt change), NS5 (P711A, K849Q)	Visible foci of GFP+ cells	28.0% (15–37%) ^4^

Notes: ^1^ Amino acid positions are numbered locally within each protein. The listed mutations originate from the adapted virus virGFP-NS1m6, published in [42]. Herein, GFP mutations are numbered according to the established eGFP nomenclature (UniProt P42212), which differs from the construct-specific numbering used in the cited work. Besides the substitution M108L in NS4A, the mutant also carries a silent nucleotide (nt) substitution, adenine to guanine, at nucleotide position 213 of the NS4B gene. ^2^ Fluorescence microscopy was performed with cultures at 72 h post-transfection and at later time points. “<1 GFP+ cell per field” means fewer than one GFP+ cell per microscopic field at an objective magnification of 5×. ^3^ The reported percentages of GFP-positive cells were determined using a gating strategy with a threshold established from the negative control, as presented in Materials and Methods (Section 2.9. Flow Cytometry Analysis) and Supplementary Figure S1. Representative flow cytometry results are shown in Supplementary Figure S2. ^4^ Presented are mean values from three biological replicates and the range (minimum–maximum).

**Table 3:** Positions of heterologous insertions into the flavivirus NS1-NS2A genes within the context of autonomously replicating RNA.

Virus	Position of the Heterologous Insertion	Distance to NS1-NS2A Junction	Tag ^1^	Viability Characteristics	Reference
YFV (17D)	In E-NS1 junction	352 a.a.	eGFP	Initially unviable. Replication-competent after adaptation	[42]
YFV (17D)	In NS1-NS2A junction	10 a.a. (these 10 a.a. from NS1 C-terminus copied after the insertion)	GFP	Initial variant non-viable. Adaptive mutations restore viability, but the adapted variant shows lower titers	This study
JEV (SD12)	NS1-NS2A junction	0 a.a. (exact junction)	HA-tag	Viable. Comparable to WT growth and plaque morphology	[39]
Dengue virus 2 (strain 16681)	In NS1, between Lys-174 and Gln-175	178 a.a. upstream of NS1-NS2A cleavage site	FLAG-tag	Viable. WT-like replication and infectious virus production	[43]
Dengue virus 2 (strain 16681)	In NS1, between Lys-174 and Gln-175	178 a.a. upstream of NS1-NS2A cleavage site	GFP11 (16 aa)	Viable. Moderately impaired	[43]
Dengue virus 2 (strain 16681)	In NS1, between Lys-174 and Gln-175	178 a.a. upstream of NS1-NS2A cleavage site	APEX2 (~28 kDa)	Viable. Attenuated. Robust replication and infectious virus production	[43]
Dengue virus 2 (strain 16681)	In NS1, between Lys-174 and Gln-175	178 a.a. upstream of NS1-NS2A cleavage site	NanoLuc/NLuc (~19 kDa)	Viable. Attenuated. Robust replication and infectious virus production	[43]
Dengue virus 2 (strain 16681)	In NS1, between Lys-174 and Gln-175	178 a.a. upstream of NS1-NS2A cleavage site	mScarlet (~27 kDa)	Viable. Supported infectious virus production despite attenuation	[43]
JEV (AT31)	In E-NS1 junction	352 a.a.	HiBiT	Viable. Comparable to wild-type virus	[45]
Chimeric vaccine against Japanese encephalitis (JE), ChimeriVax-JE	In NS1, between Val-236 and Glu-237	179 a.a. upstream of NS1-NS2A cleavage site	M2e peptide of influenza A virus (35 a.a.)	Viable. No significant effect on virus replication	[47]

Notes: ^1^ Epitope tags: HA-tag (YPYDVPDYA), FLAG-tag (DYKDDDDK); APEX2, peroxidase or engineered ascorbate peroxidase 2; NanoLuc, luciferase enzyme; mScarlet, monomeric red fluorescent protein; HiBiT, 11 a.a. peptide from the NanoLuc luciferase.

## Data Availability

Data is contained within the article or Supplementary Material. Dataset available on request from the authors.

## Supplementary Materials

The following supporting information can be downloaded at: https://www.mdpi.com/article/10.3390/biology15030220/s1. Figure S1: Gating strategy for flow cytometry analysis of GFP-positive cells; Figure S2: Representative flow cytometry dot plots; Figure S3: Scan of an entire membrane for Western blot analysis of intracellular proteins (related to Figure 7); Figure S4: Complete Western blot membrane for the subcellular fractionation analysis (related to Figure 8a); Figure S5: Complete Coomassie-stained gel image of the heavy membrane flotation gradient (related to Figure 8b); Figure S6: Complete Western blot membrane of the flotation gradient fractions (related to Figure 8c); Figure S7: Mass spectrometric analysis confirms the presence of the NS1-GFP protein in the heavy membrane gradient fraction.

## Abbreviations

The following abbreviations are used in this manuscript:

BSL-2	Biosafety level 2
CLEM	Correlative light and electron microscopy
CMV	Cytomegalovirus
cryo-ET	Cryo-electron tomography
DAPI	4′,6-diamidino-2-phenylindole
DMEM	Dulbecco’s Modified Eagle Medium
ER	Endoplasmic reticulum
FBS	Fetal Bovine Serum
FFU	Focus-forming unit
FIB-SEM	Focused ion beam scanning electron microscopy
GFP	Green fluorescent protein
HDV-Rz	Hepatitis delta virus antigenomic ribozyme
HGH	Human growth hormone
MIC	Molecular infectious clone
MOI	Multiplicity of infection
PA	Polyadenylation signal
Pac	Puromycin acetyltransferase
PBS	Phosphate-buffered saline
RC	Replication complex
RdRp	RNA-dependent RNA polymerase
RO	Replication organelle
SP	Subgenomic promoter
UTR	Untranslated region
VEEV	Venezuelan equine encephalitis virus
YFV	Yellow fever virus

## References

[1] Postler T.S., Beer M., Blitvich B.J., Bukh J., de Lamballerie X., Drexler J.F., Imrie A., Kapoor A., Karganova G.G., Lemey P. (2023). Renaming of the genus *Flavivirus* to *Orthoflavivirus* and extension of binomial species names within the family *Flaviviridae*. Arch. Virol..

[2] Parry R., Asgari S. (2019). Discovery of novel crustacean and cephalopod flaviviruses: Insights into the evolution and circulation of flaviviruses between marine invertebrate and vertebrate hosts. J. Virol..

[3] Lensink M.J., Li Y., Lequime S. (2022). Aquatic flaviviruses. J. Virol..

[4] Dong X., Meng F., Zhou C., Li J., Hu T., Wang Y., Wang G., Luo J., Li X., Liu S. (2024). Enormous diversity of RNA viruses in economic crustaceans. mSystems.

[5] Pierson T.C., Diamond M.S. (2020). The continued threat of emerging flaviviruses. Nat. Microbiol..

[6] Harris E., Holden K.L., Edgil D., Polacek C., Clyde K. (2006). Molecular biology of flaviviruses. New Treatment Strategies for Dengue and Other Flaviviral Diseases.

[7] Barrows N.J., Campos R.K., Liao K.-C., Prasanth K.R., Soto-Acosta R., Yeh S.-C., Schott-Lerner G., Pompon J., Sessions O.M., Bradrick S.S. (2018). Biochemistry and molecular biology of flaviviruses. Chem. Rev..

[8] van den Elsen K., Quek J.P., Luo D. (2021). Molecular insights into the flavivirus replication complex. Viruses.

[9] Donaldson M.K., Zanders L.A., Jose J. (2025). Functional roles and host interactions of orthoflavivirus non-structural proteins during replication. Pathogens.

[10] Dey D., Poudyal S., Rehman A., Hasan S.S. (2021). Structural and biochemical insights into flavivirus proteins. Virus Res..

[11] Zhang S., He Y., Wu Z., Wang M., Jia R., Zhu D., Liu M., Zhao X., Yang Q., Wu Y. (2023). Secretory pathways and multiple functions of nonstructural protein 1 in flavivirus infection. Front. Immunol..

[12] Muller D.A., Young P.R. (2013). The flavivirus NS1 protein: Molecular and structural biology, immunology, role in pathogenesis and application as a diagnostic biomarker. Antivir. Res..

[13] Akey D.L., Brown W.C., Dutta S., Konwerski J., Jose J., Jurkiw T.J., DelProposto J., Ogata C.M., Skiniotis G., Kuhn R.J. (2014). Flavivirus NS1 structures reveal surfaces for associations with membranes and the immune system. Science.

[14] Luo D., Vasudevan S.G., Lescar J. (2015). The flavivirus NS2B–NS3 protease-helicase as a target for antiviral drug development. Antivir. Res..

[15] Erbel P., Schiering N., D’Arcy A., Renatus M., Kroemer M., Lim S.P., Yin Z., Keller T.H., Vasudevan S.G., Hommel U. (2006). Structural basis for the activation of flaviviral NS3 proteases from dengue and West Nile virus. Nat. Struct. Mol. Biol..

[16] Luo D., Xu T., Watson R.P., Scherer-Becker D., Sampath A., Jahnke W., Yeong S.S., Wang C.H., Lim S.P., Strongin A. (2008). Insights into RNA unwinding and ATP hydrolysis by the flavivirus NS3 protein. EMBO J..

[17] Goh J.Z., De Hayr L., Khromykh A.A., Slonchak A. (2024). The flavivirus non-structural protein 5 (NS5): Structure, functions, and targeting for development of vaccines and therapeutics. Vaccines.

[18] Klaitong P., Smith D.R. (2021). Roles of non-structural protein 4A in flavivirus infection. Viruses.

[19] Wang Y., Xie X., Shi P.Y. (2022). Flavivirus NS4B protein: Structure, function, and antiviral discovery. Antivir. Res..

[20] Leung J.Y., Pijlman G.P., Kondratieva N., Hyde J., Mackenzie J.M., Khromykh A.A. (2008). Role of nonstructural protein NS2A in flavivirus assembly. J. Virol..

[21] Mackenzie J.M., Jones M.K., Westaway E.G. (1999). Markers for trans-Golgi membranes and the intermediate compartment localize to induced membranes with distinct replication functions in flavivirus-infected cells. J. Virol..

[22] Arakawa M., Morita E. (2019). Flavivirus replication organelle biogenesis in the endoplasmic reticulum. Int. J. Mol. Sci..

[23] Ci Y., Shi L. (2021). Compartmentalized replication organelle of flavivirus at the ER and the factors involved. Cell. Mol. Life Sci..

[24] Assenberg R., Mastrangelo E., Walter T.S., Verma A., Milani M., Owens R.J., Stuart D.I., Grimes J.M., Mancini E.J. (2009). Crystal structure of a novel conformational state of the flavivirus NS3 protein. J. Virol..

[25] Wahaab A., Mustafa B.E., Hameed M., Stevenson N.J., Anwar M.N., Liu K., Wei J., Qiu Y., Ma Z. (2021). Potential role of flavivirus NS2B–NS3 proteases in viral pathogenesis and drug discovery. Viruses.

[26] Alzahrani N., Wu M.J., Shanmugam S., Yi M. (2020). Delayed by design: Role of suboptimal signal peptidase processing of viral structural protein precursors. Viruses.

[27] Pierson T.C., Diamond M.S. (2012). Degrees of maturity: The complex structure and biology of flaviviruses. Curr. Opin. Virol..

[28] Apte-Sengupta S., Sirohi D., Kuhn R.J. (2014). Coupling of replication and assembly in flaviviruses. Curr. Opin. Virol..

[29] Addis S.N., Lee E., Bettadapura J., Lobigs M. (2015). Proteolytic cleavage analysis at the Murray Valley encephalitis virus NS1–2A junction. Virol. J..

[30] Blitvich B.J., Scanlon D., Shiell B.J., Mackenzie J.S., Hall R.A. (1999). Identification and analysis of truncated and elongated species of the flavivirus NS1 protein. Virus Res..

[31] Pethel M., Falgout B., Lai C.J. (1992). Mutational analysis of the octapeptide motif at the NS1–NS2A cleavage junction of dengue virus type 4. J. Virol..

[32] Youn S., Ambrose R.L., Mackenzie J.M., Diamond M.S. (2013). Nonstructural protein 1 is required for West Nile virus replication complex formation. Virol. J..

[33] Lindenbach B.D., Rice C.M. (1999). Genetic interaction of flavivirus NS1 and NS4A proteins. J. Virol..

[34] Xu X., Gubler D.J., Yang Z., Shi P.Y. (2013). Membrane topology and function of dengue virus NS2A protein. J. Virol..

[35] Shan C., Xie X., Zou J., Züst R., Zhang B., Ambrose R., Mackenzie J., Fink K., Shi P.-Y. (2018). Using a virion assembly-defective dengue virus as a vaccine approach. J. Virol..

[36] Zhang X., Xie X., Xia H., Zou J., Huang L., Popov V.L., Chen X., Shi P.-Y. (2019). Zika virus NS2A-mediated virion assembly. mBio.

[37] Xie X., Zou J., Zhang X., Zhou Y., Routh A.L., Kang C., Popov V.L., Chen X., Wang Q.-Y., Dong H. (2019). Dengue NS2A protein orchestrates virus assembly. Cell Host Microbe.

[38] Zeidler J.D., Fernandes-Siqueira L.O., Barbosa G.M., Da Poian A.T. (2017). Non-canonical roles of dengue virus non-structural proteins. Viruses.

[39] Ma X., Li C., Xia Q., Zhang Y., Yang Y., Wahaab A., Liu K., Li Z., Li B., Qiu Y. (2022). Construction of a recombinant Japanese encephalitis virus with hemagglutinin-tagged NS2A. Viruses.

[40] Branon T.C., Bosch J.A., Sanchez A.D., Udeshi N.D., Svinkina T., Carr S.A., Feldman J.L. (2018). Efficient proximity labeling in living cells with TurboID. Nat. Biotechnol..

[41] Roux K.J., Kim D.I., Raida M., Burke B. (2012). A promiscuous biotin ligase identifies proximal proteins in mammalian cells. J. Cell Biol..

[42] Syzdykova L.R., Binke S., Keyer V.V., Shevtsov A.B., Zaripov M.M., Zhylkibayev A.A., Ramanculov E.M., Shustov A.V. (2021). Fluorescent tagging of NS1 protein in yellow fever virus. Virus Res..

[43] Eyre N.S., Johnson S.M., Eltahla A.A., Aloi M., Aloia A.L., McDevitt C.A., Bull R.A., Beard M.R. (2017). Genome-wide mutagenesis of dengue virus NS1. J. Virol..

[44] Mason P.W., Shustov A.V., Frolov I. (2006). Production and characterization of vaccines based on flaviviruses defective in replication. Virology.

[45] Tamura T., Fukuhara T., Uchida T., Ono C., Mori H., Sato A., Fauzyah Y., Okamoto T., Kurosu T., Setoh Y.X. (2018). Characterization of recombinant *Flaviviridae* viruses possessing a small reporter tag. J. Virol..

[46] Tamura T., Igarashi M., Enkhbold B., Suzuki T., Okamatsu M., Ono C., Mori H., Izumi T., Sato A., Fauzyah Y. (2019). In vivo dynamics of reporter *Flaviviridae* viruses. J. Virol..

[47] Rumyantsev A.A., Zhang Z.X., Gao Q.S., Moretti N., Brown N., Kleanthous H., Delagrave S., Guirakhoo F., Collett M.S., Pugachev K.V. (2010). Direct random insertion of an influenza virus determinant into flavivirus NS1. Virology.

[48] Youn S., Li T., McCune B.T., Edeling M.A., Fremont D.H., Cristea I.M., Diamond M.S. (2012). Interaction between NS1 and NS4B modulates West Nile virus replication. J. Virol..

[49] Tan M.J., Brown N.G., Chan K.W., Jin J.Y., Kong S.Y., Vasudevan S.G. (2020). Mutations in dengue virus NS4A affect virus fitness. J. Gen. Virol..

[50] Zacharias D.A., Violin J.D., Newton A.C., Tsien R.Y. (2002). Partitioning of lipid-modified monomeric GFPs into membrane microdomains. Science.

[51] von Stetten D., Noirclerc-Savoye M., Goedhart J., Gadella T.W.J., Royant A. (2012). Structure of a fluorescent protein bearing the A206K mutation. Acta Crystallogr. F.

[52] Roberts T.M., Rudolf F., Meyer A., Pellaux R., Whitehead E., Panke S., Held M. (2016). Identification and characterisation of a pH-stable GFP. Sci. Rep..

