# Supplementary material for: A Replication-Competent Flavivirus Genome with a Stable GFP Insertion at the NS1-NS2A Junction
Source: Biology (Basel). 2026 Jan 24;15(3):220. doi: 10.3390/biology15030220 (PMC12896631; doi:10.3390/biology15030220)
Supplement: Supplementary file 1 [file biology-15-00220-s001.zip › biology-4082788-supplementary.pdf]

Supplementary material to

A replication-competent flavivirus genome with a stable GFP insertion at the NS1-NS2A junction

Pavel Tarlykov [1], Bakytkali Ingirbay [1], Dana Auganova [1], Tolganay Kulatay [1], Viktoriya Keyer [1], Sabina Atavliyeva [1], Maral Zhumabekova [1], Arman Abeev [1], Alexandr V. Shustov [1,*]

[1] National Center for Biotechnology, Korgalzhin hwy 13/5, 010000, Astana, Kazakhstan

* Correspondence: shustov@biocenter.kz , Tel: +77024735305

P.T.	Pavel Tarlykov	ORCID 0000-0003-2075-307X	tarlykov@biocenter.kz
B.I.	Bakytkali Ingirbay	ORCID 0000-0002-6915-8207	ingirbay@biocenter.kz
D.A.	Dana Auganova	ORCID 0000-0002-5719-6118	auganova@biocenter.kz
T.K.	Tolganay Kulatay	ORCID 0009-0004-5885-8963	kulatay@biocenter.kz
V.K.	Viktoriya Keyer	ORCID 0000-0001-8885-2387	keer@biocenter.kz
S.A.	Sabina Atavliyeva	ORCID 0000-0002-7565-9454	atavliyeva@biocenter.kz
M.Zh.	Maral Zhumabekova	ORCID 0009-0002-9532-6232	zhumabekova@biocenter.kz
A.A.	Arman Abeev	ORCID 0009-0005-3594-425X	abeev@biocenter.kz
A.V.S.	Alexandr V. Shustov	ORCID 0000-0001-9880-9382	shustov@biocenter.kz

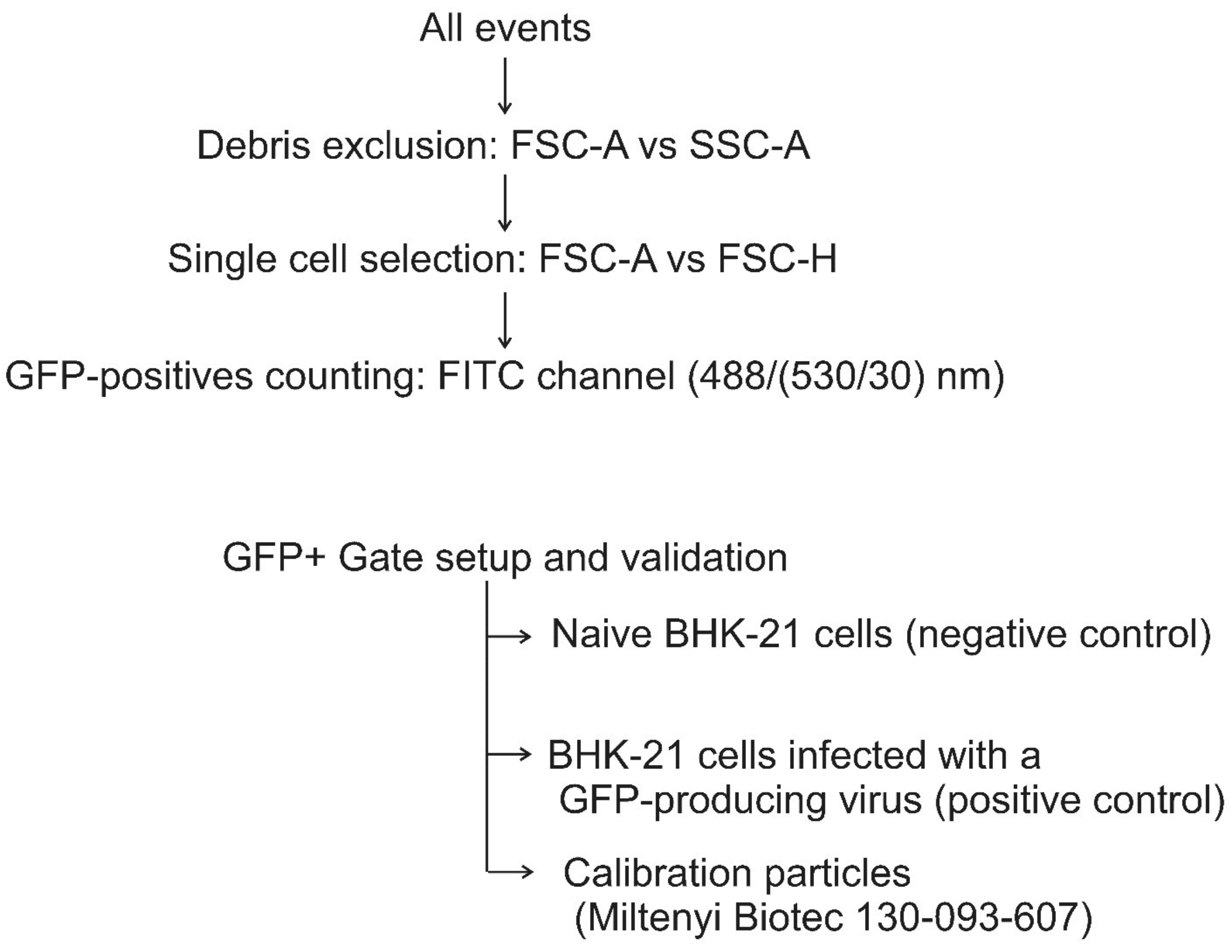

Figure S1. Gating strategy for quantifying GFP-positive cells in cultures transfected with GFP-expressing YFV-derived constructs.

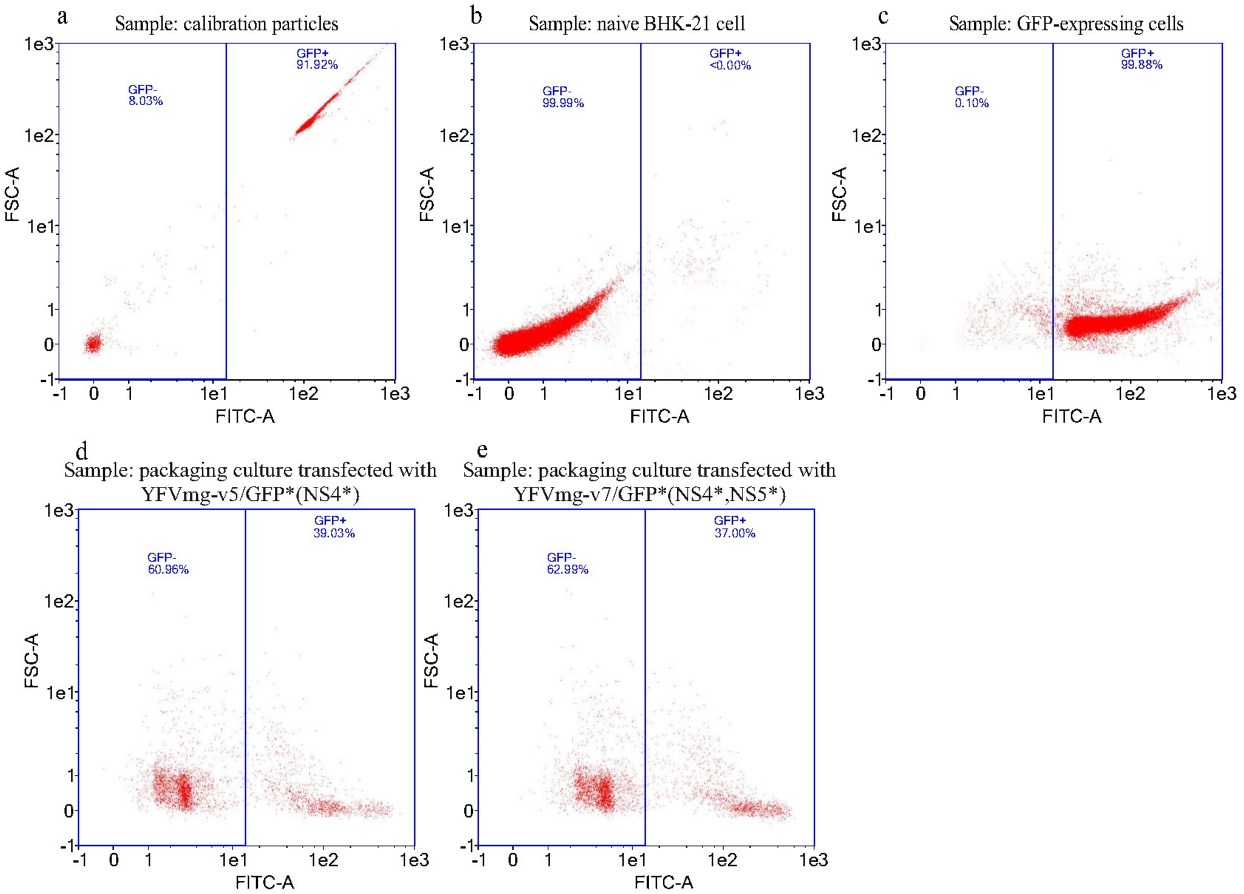

Figure S2. Flow cytometry dot plots for determining the percentage of GFP-positive (GFP+) cells.

(a) Fluorescent calibration particles (Miltenyi Biotec) were used to establish flow cytometry gates for GFP+ and GFP-negative (GFP-) populations.

(b) Naïve BHK-21 cells were analyzed to validate the GFP- gate. Less than 0.01% of events remained in the positive gate.

(c) BHK-21 cells infected with a fast-growing YFV-derived GFP-expressing virus were used to validate the GFP+ gate. High multiplicity of infection (MOI = 10) was used to infect nearly all cells in the culture; flow cytometry was performed at 36 hours post-infection before the cytopathic effect was evident. 99.88% of events were counted within the GFP+ gate.

(d) Flow cytometry analysis of GFP expression in packaging cells (producing yellow fever virus structural proteins C-prM-E) transfected with the minigenome YFVmg-v5/GFP*(NS4*).

(e) Flow cytometry analysis of GFP expression in packaging cells (+C-prM-E) transfected with the minigenome YFVmg-v7/GFP*(NS4*,NS5*).

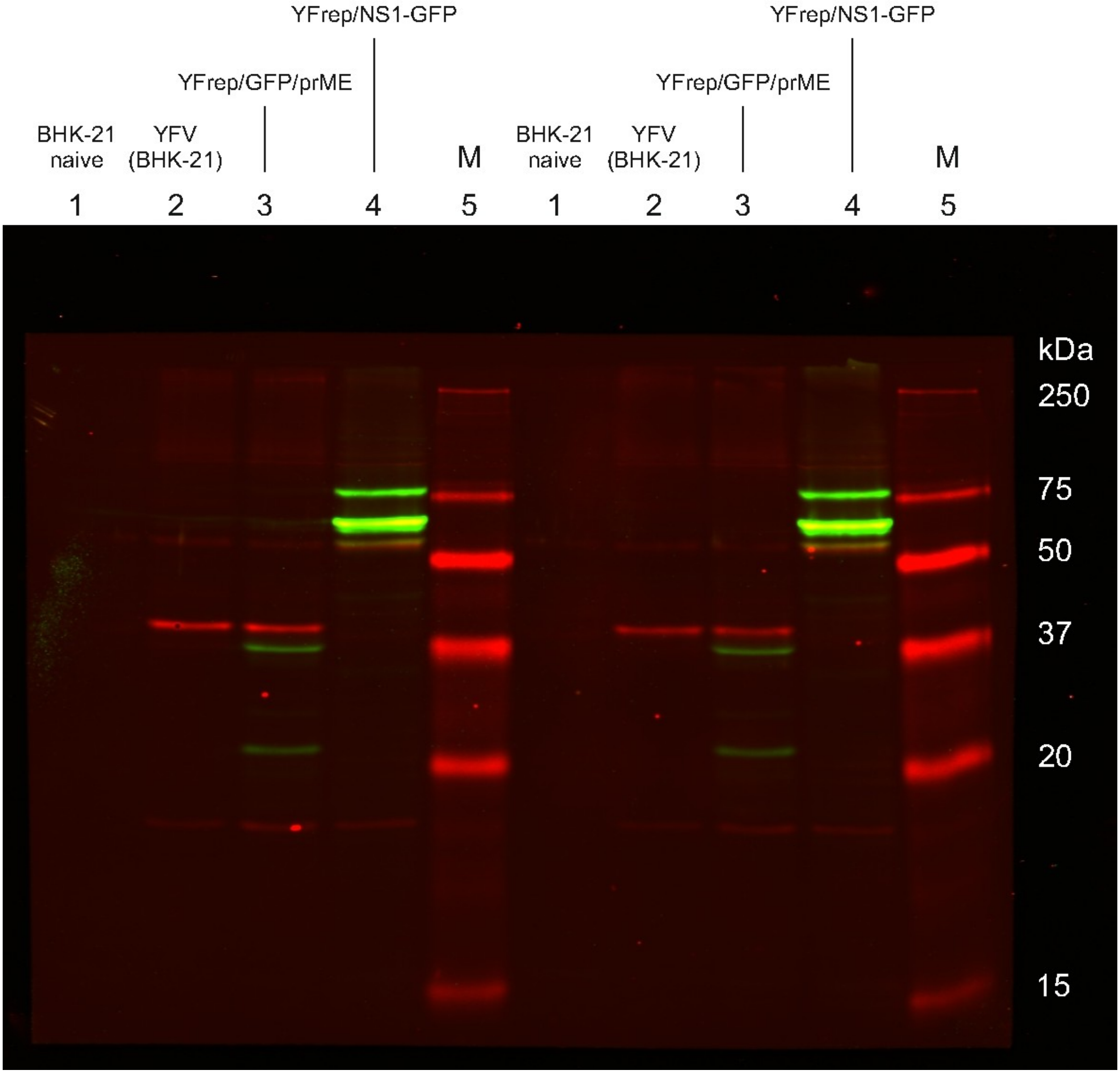

Figure S3. Scan of an entire membrane for western blot analysis of intracellular proteins in cells harboring YFV or YFV-derived replicons (related to Figure 7). The figure shows a two-color immunoblot of cell lysates stained with a polyclonal mouse anti-YFV antiserum (red channel, 700 nm) and an anti-GFP antibody (green channel, 800 nm). For the Western blot, samples were loaded onto a single gel as two identical series. Only half of the membrane is shown in Figure 7 (main text). Imaging was performed on a LI-COR Odyssey system. Lane designations: 1, Naive BHK-21 cells (uninfected control). 2, BHK-21 cells infected with wild-type YFV. 3, Packaging cells harboring the YFrep/GFP/prME replicon, expressing the 25C-GFP-2A fusion protein. 4, Packaging cells harboring the YFrep/NS1-GFP replicon, expressing the NS1-GFP fusion protein. 5, Protein marker (Precision Plus Protein Kaleidoscope, Bio-Rad #1610375); only marker bands detected in the 700 nm channel are visible. Molecular weights (in kDa) of the protein marker are indicated on the right. Key findings: YFV-specific proteins (red bands) detected in lanes 2-4: NS5 (104 kDa), NS3 (69.2 kDa), NS1 (39.7 kDa), and prM (18.7 kDa). GFP-containing fusion proteins (green bands) detected in lanes 3 and 4.

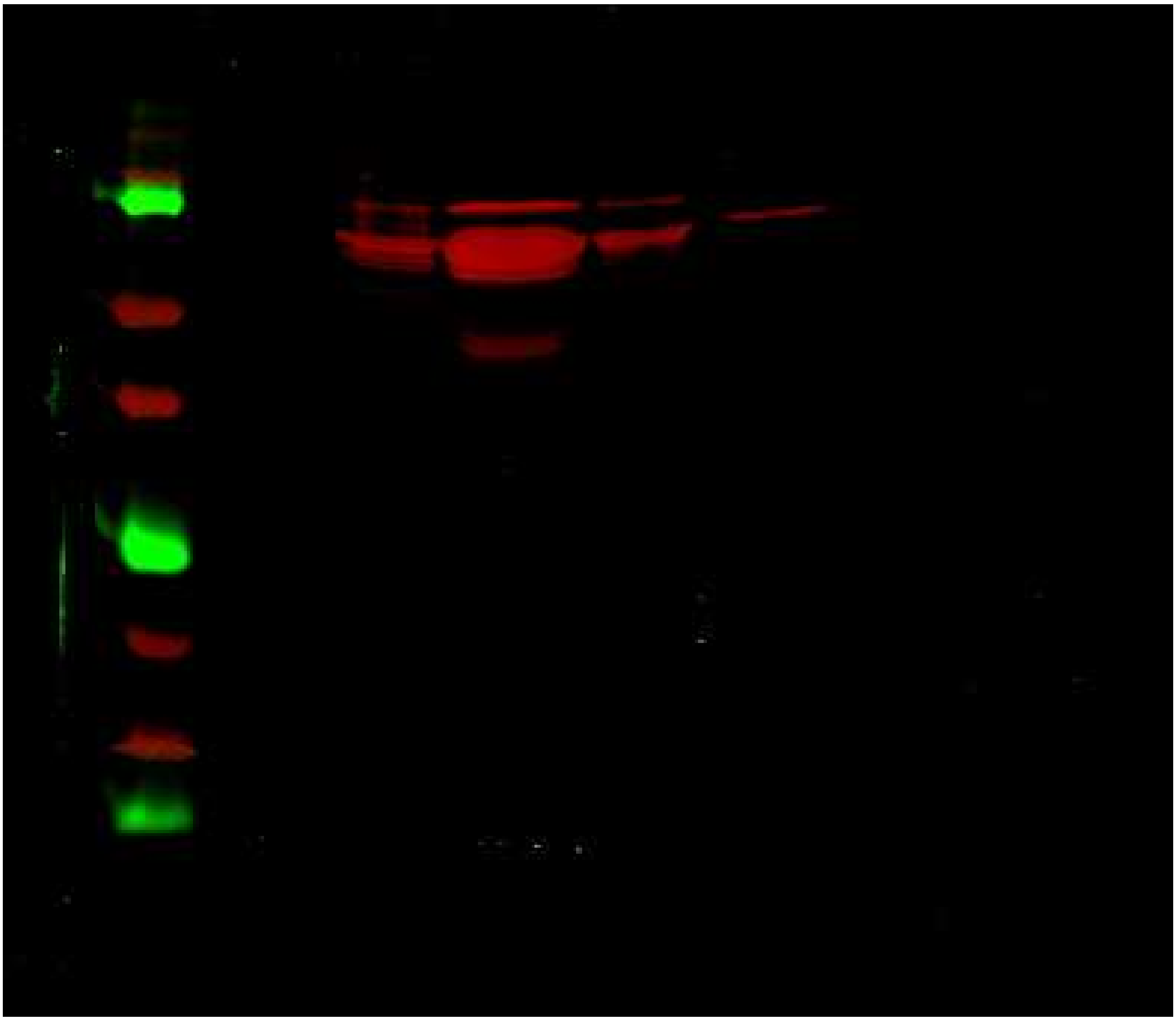

Figure S4. Complete western blot membrane for the subcellular fractionation analysis (related to Figure 8a). The photograph shows the two-color immunoblot of subcellular fractions probed with an anti-NS1 antibody (red channel, 700 nm). Imaging was performed on a LI-COR Odyssey system. Samples were loaded as follows (left to right): 1, Precision Plus Protein Kaleidoscope marker (Bio-Rad, Cat. 1610375); 2, whole-cell lysate of naïve BHK-21 cells (uninfected control); 3, nuclear fraction (P720, pellet after centrifugation at 720 × g); 4, heavy membrane fraction (P16K, pellet after centrifugation at 16,000 × g); 5, post-mitochondrial supernatant (S16K, supernatant after centrifugation at 16,000 × g); 6, light membranes (P35K, pellet after ultracentrifugation (35 000 × g) of the S16K fraction).

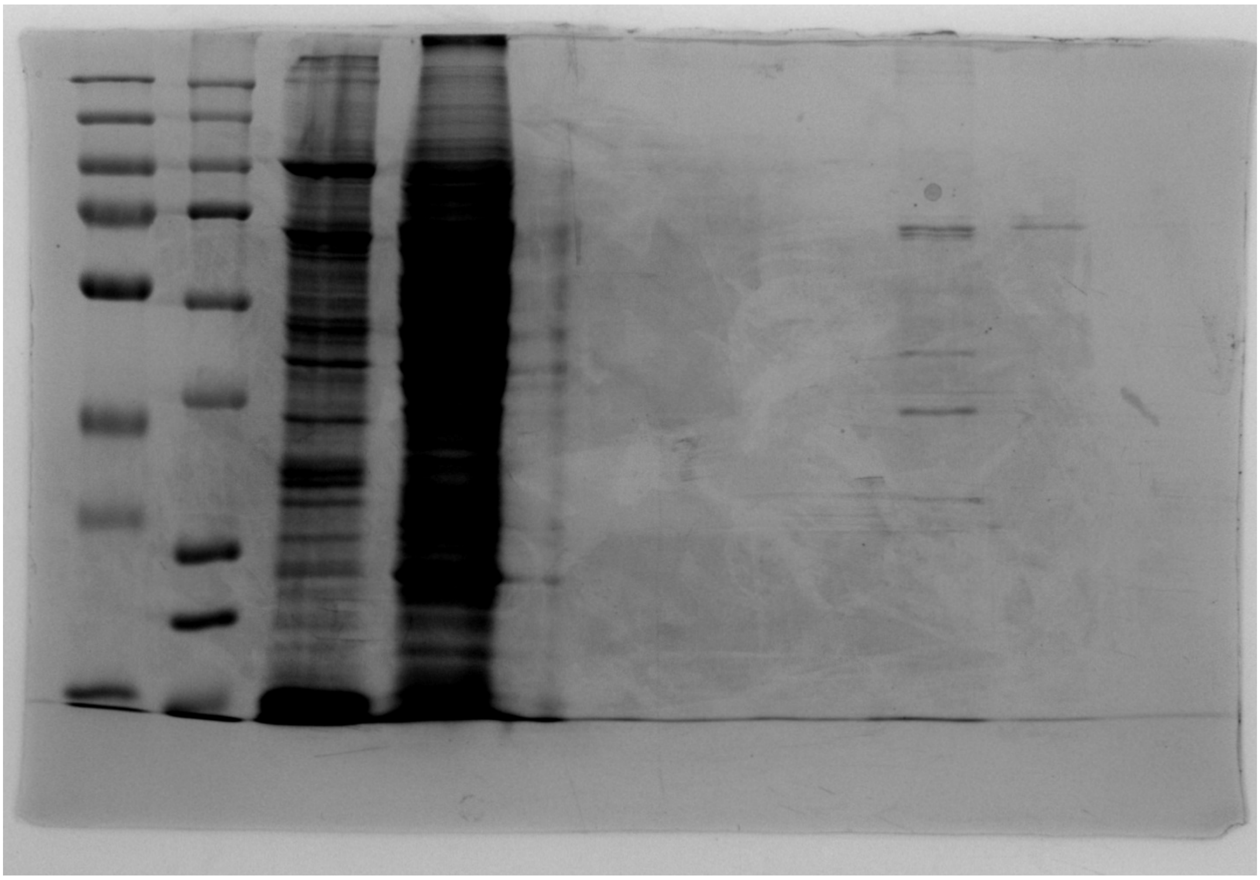

Figure S5. Photograph of the gel from the SDS-PAGE analysis of a heavy membrane fraction from infected cells; a portion of the gel is shown in Figure 8b. Samples were loaded as follows (left to right), lanes: 1, PageRuler Plus 10-250 kDa (Thermo Scientific, Cat. 26619); 2, Precision Plus Protein Kaleidoscope marker (Bio-Rad, Cat. 1610375); 3, P720, nuclei pellet after 720 × g); 4, S16K, cytosol and light membranes (supernatant after 16,000 × g); 5-10, gradient fractions 1-6 upon separation of heavy membranes, P16K (pellet after 16,000 × g).

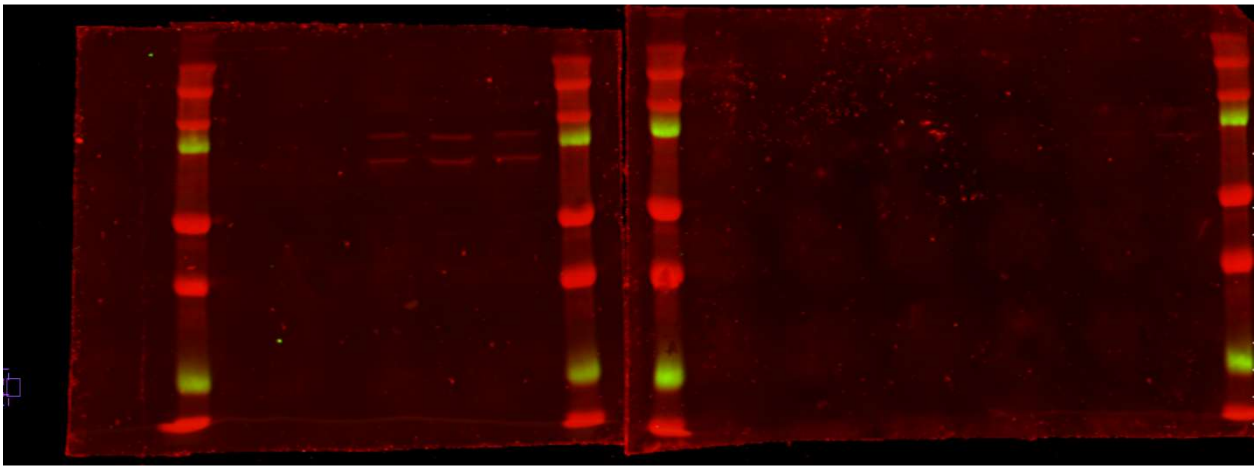

Figure S6. Complete western blot membranes from the flotation gradient centrifugation analysis (related to Figure 8c).

The photograph shows two membranes that were scanned side-by-side. Both contain samples from the 13 fractions collected after flotation gradient centrifugation of the P16K heavy membrane fraction (top to bottom). Proteins were separated by SDS-PAGE, transferred to nitrocellulose, and probed with an anti-NS1 monoclonal antibody.

Left membrane: Lanes were loaded as follows (left to right):

1, Precision Plus Protein Kaleidoscope marker (Bio-Rad, Cat. 1610375).

2–6, gradient fractions 1–5 (top of the gradient to mid-gradient).

7, Precision Plus Protein Kaleidoscope marker.

Right membrane: Lanes were loaded as follows (left to right):

1, Precision Plus Protein Kaleidoscope marker.

2–9, gradient fractions 6–13 (mid-gradient to bottom of the gradient).

10, Precision Plus Protein Kaleidoscope marker.

MATRIX SCIENCE MASCOT Search Results

Protein View: NS1-GFP

NS1-GFP

Database:	YFrep/NS1-GFP_all_proteins
Score:	1402
Monoisotopic mass (Mr):	68389
Calculated pI:	5.92

Sequence similarity is available as an NCBI BLAST search of NS1-GFP against YFrep/NS1-GFP_all_proteins

Search parameters

MS data file:	ProteinAnalysisResults.mgf
Enzyme:	Trypsin: cuts C-term side of KR unless next residue is P.
Fixed modifications:	Carbamidomethyl (C)
Variable modifications:	Oxidation (M)

Protein sequence coverage: 14%

Matched peptides shown in ***bold red***

1	DQGCAINFGK	RELK**CGDGIF**	**IFR**DSDDWLN	KYSYYPEDPV	KLASIVKASF
51	EEGKCGLNSV	DSLEHEMWRS	RADEINAIFE	ENEVDISVVV	QDPKNVYQRG
101	THPFSRIRDG	LQYGWKTWGK	**NLVFSPGR**KN	GSFIIDGKSR	KECPFSNRVW
151	NSFQIEEFGT	GVFTTRVYMD	AVFEYTIDCD	GSILGAAVNG	KKSAHGSPTF
201	WMGSHEVNGT	WMIHTLEALD	YKECEWPLTH	TIGTSVEESE	MFMPRSIGGP
251	VSSHNHIPGY	K**VQTNGPWMQ**	**VPLEVKREAC**	**PGTSVIIDGN**	**CDGR**GKSTRS
301	TTDSGKVIPE	WCCRSCTMPP	VSFHGSDGCW	YPMEIRPR**KT**	**HESHLVR**SWV
351	TAG**SGPGSVV**	**SK**GEELFTGV	VPILVELDGD	VNGHKFSVSG	EGEGDATYGK
401	LTLKFICTTG	KLPVPWPTLV	TTLTYGVQCF	SRYPDHMKQH	DFFKSAMPEG
451	YVQERTIFFK	DDGNYKTRAE	VKFEGDTLVN	RIELKGIDFK	EDGNILGHKL
501	EYNYN**SHNVY**	**IMADK**QKNGI	KVNFKIRHNI	EDGSVQLADH	YQQNTPIGDG
551	PVLLPDNHYL	**STQSALSK**DP	NEKRDHMVLL	EFVTAAGITL	GMDELYKSRS
601	HLVRSWVTA				

Figure S7. Mass spectrometric analysis confirms the presence of the NS1-GFP protein in the heavy membrane gradient fraction. The main band indicated by the green arrow in Figure 8a, was excised and subjected to mass-spectrometric identification of tryptic peptides.
